# Antibody to Poly-*N*-acetyl glucosamine provides protection against intracellular pathogens: Mechanism of action and validation in horse foals challenged with *Rhodococcus equi*

**DOI:** 10.1371/journal.ppat.1007160

**Published:** 2018-07-19

**Authors:** Colette Cywes-Bentley, Joana N. Rocha, Angela I. Bordin, Mariana Vinacur, Safia Rehman, Tanweer S. Zaidi, Mark Meyer, Sarah Anthony, McKenzie Lambert, Daniel R. Vlock, Steeve Giguère, Noah D. Cohen, Gerald B. Pier

**Affiliations:** 1 Harvard Medical School, Brigham & Women’s Hospital, Boston, MA, United States of America; 2 College of Veterinary Medicine & Biomedical Sciences, Texas A&M University, College Station, TX, United States of America; 3 Mg Biologics, Ames, IA, United States of America; 4 ALOPEXX Vaccine LLC, Concord, MA, United States of America; 5 College of Veterinary Medicine, University of Georgia, Athens, GA, United States of America; University of Washington, UNITED STATES

## Abstract

Immune correlates of protection against intracellular bacterial pathogens are largely thought to be cell-mediated, although a reasonable amount of data supports a role for antibody-mediated protection. To define a role for antibody-mediated immunity against an intracellular pathogen, *Rhodococcus equi*, that causes granulomatous pneumonia in horse foals, we devised and tested an experimental system relying solely on antibody-mediated protection against this host-specific etiologic agent. Immunity was induced by vaccinating pregnant mares 6 and 3 weeks prior to predicted parturition with a conjugate vaccine targeting the highly conserved microbial surface polysaccharide, poly-*N*-acetyl glucosamine (PNAG). We ascertained antibody was transferred to foals via colostrum, the only means for foals to acquire maternal antibody. Horses lack transplacental antibody transfer. Next, a randomized, controlled, blinded challenge was conducted by inoculating at ~4 weeks of age ~10^6^ cfu of *R*. *equi* via intrabronchial challenge. Eleven of 12 (91%) foals born to immune mares did not develop clinical *R*. *equi* pneumonia, whereas 6 of 7 (86%) foals born to unvaccinated controls developed pneumonia (P = 0.0017). In a confirmatory passive immunization study, infusion of PNAG-hyperimmune plasma protected 100% of 5 foals against *R*. *equi* pneumonia whereas all 4 recipients of normal horse plasma developed clinical disease (P = 0.0079). Antibodies to PNAG mediated killing of extracellular and intracellular *R*. *equi* and other intracellular pathogens. Killing of intracellular organisms depended on antibody recognition of surface expression of PNAG on infected cells, along with complement deposition and PMN-assisted lysis of infected macrophages. Peripheral blood mononuclear cells from immune and protected foals released higher levels of interferon-γ in response to PNAG compared to controls, indicating vaccination also induced an antibody-dependent cellular release of this critical immune cytokine. Overall, antibody-mediated opsonic killing and interferon-γ release in response to PNAG may protect against diseases caused by intracellular bacterial pathogens.

## Author summary

Development of effective vaccines for diseases such as tuberculosis, brucellosis and others caused by intracellular pathogens has proved challenging, as data exist supporting both antibody and cellular immune effectors as mediators of protection. To address this problem against an important, and representative, equine intracellular pathogen, we chose to test a vaccine candidate for the ability to protect horse foals challenged at 4 weeks of age with *Rhodococcus equi*. To make these foals immune, their pregnant mares were immunized with a vaccine targeting the conserved surface antigen found on many microbes, termed PNAG. Antibody in the pregnant mares was transferred to their foals and, after the foals were challenged, 91% of those born to vaccinated mares were protected against *R*. *equi* pneumonia. Meanwhile, 86% of the non-vaccinated controls developed pneumonia. We also showed antibody to PNAG could kill various bacteria that produce this antigen when residing inside of human macrophage cells, a new mechanism for antibody-mediated immunity to intracellular bacteria. These results support the development of PNAG as a vaccine for intracellular bacterial pathogens.

## Introduction

Correlates of cellular and humoral immunity to major intracellular, non-viral pathogens capable of informing vaccine development are incompletely understood. It is unknown which ones can form the basis of a highly effective vaccine to prevent diseases such as tuberculosis (TB). Protection studies conducted to date, primarily in laboratory rodents and non-human primates, have not led to an effective human vaccine for such pathogens [1, 2] outside of the limited efficacy of the live Bacillus Calmette-Guerin whole-cell vaccine against TB [2–4]. *Rhodococcus equi* is a Gram-positive, facultative intracellular pathogen carrying an essential virulence plasmid that primarily infects alveolar macrophages of horse foals following inhalation. *R*. *equi* replicates within a modified phagocytic vacuole, with survival dependent on the virulence plasmid preventing phagosome-lysosome fusion, resulting in a granulomatous pneumonia that is pathologically similar to that caused by *Mycobacterium tuberculosis* infection in humans [5]. *R*. *equi* also causes extrapulmonary disorders including osseous and intra-abdominal lymphadenitis [5–7]. The disease is of considerable importance to the equine industry [5, 7], and while some reports indicate vaccination and/or passive transfer of hyperimmune plasma using bactrin-based or virulence associated protein A vaccines can reduce the severity of *R*. *equi* pneumonia [8, 9], it is generally felt that most attempts to date to create an effective *R*. *equi* vaccine have been unsuccessful [10, 11]. There is no approved vaccine for *R*. *equi* in any animal species.

Presently, it can be solidly reasoned that cell-mediated immune (CMI) responses underlay the basis for natural immunity to *R*. *equi*. Disease occurs almost exclusively in foals less than 6 months of age, but by ~9 months of age most young horses become highly resistant to this pathogen [5–7, 12]. This acquired natural resistance is obviously not antibody-mediated inasmuch as the solid immunity to infection in healthy horses >9 months of age, which obviously includes pregnant mares, is not transferred to susceptible foals via antibody in the colostrum. Colostrum is the only source of maternal antibody in foals and the offspring of other animals producing an epitheliochorial placenta. Therefore, an effectual vaccine trial can be designed to test whether an antibody-eliciting immunogen is efficacious by immunization of pregnant mares, that should lead to colostral transfer of vaccine-induced antibody to their offspring, with a subsequent evaluation of protective efficacy following challenge of these foals with virulent *R*. *equi*.

*R*. *equi* synthesizes the conserved surface capsule-like polysaccharide, poly-*N*-acetyl glucosamine (PNAG), wherein this antigen is intercalated into the same extracellular space as classical bacterial capsules [13] or serves as a single, encapsulating antigen on the surface of organisms such as *Neisseria gonorrhoeae* and non-typable *Hemophilus influenzae* [13]. PNAG is also expressed by fungal and protozoan pathogens [13]. As such, PNAG is a target for the development of a vaccine potentially protective against many pathogens [13, 14]. Since numerous microbes produce this antigen, there is natural IgG antibody in most human and animal sera [15, 16]. But natural antibody is generally ineffective at eliciting protection against infection. Natural antibodies usually poorly activate the complement pathway and thus ineffectively mediate microbial killing [15–17]. By removing most of the acetate substituents from the *N*-acetyl glucosamine sugars comprising PNAG [18, 19], or using synthetic oligosaccharides composed of only β-1→6-linked glucosamine conjugated to a carrier protein such as tetanus toxoid (TT), [13, 17, 20, 21] complement-fixing, microbial-killing, and protective antibody to PNAG can be induced. A final premise justifying immunizing pregnant mares to evaluate vaccine-induced immunity to *R*. *equi* is that foals are considered to be infected soon after birth [22] when they are more susceptible to infection [23] and when their immune system is less effective in responding to vaccines [10, 24–26]. This precludes active immunization of very young foals as a strategy for vaccine evaluation against *R*. *equi*. Indeed, as part of our clinical evaluations of a PNAG vaccine for *R*. *equi*, we attempted to immunize foals starting at two days of age and were unsuccessful at inducing antibody. Therefore, in order to ascertain if *R*. *equi* pneumonia could be prevented by antibody to PNAG, pregnant mares were vaccinated with the 5GlcNH_2_-TT vaccine, the transfer of functional opsonic antibodies via colostrum to foals verified, and foals were challenged at 25–28 days of life with virulent *R*. *equi*. The primary hypothesis of this randomized, controlled, blinded challenge study was that induction of complement-fixing, functional antibody to PNAG would prevent the development of clinical *R*. *equi* pneumonia in challenged foals.

## Results

### Maternal vaccination induces serum and colostral antibody to PNAG that is orally transferred to foals

Mares were immunized twice approximately 6 and 3 weeks prior to their estimated date of parturition (based on last known breeding date) with 125 or 200 μg of the 5GIcNH_2_ vaccine conjugated to TT (AV0328 from Alopexx Vaccine, LLC) adjuvanted with 100 μl of Specol. Immunization of mares resulted in no detectable local or systemic reaction following either 1 or 2 vaccine doses except for a slightly swollen muscle 24 h after the first vaccination followed at day 2 by a small dependent edema that resolved by day 3 in a single mare. Serum samples from mares immunized in 2015 were only collected on the day of foaling, so statistical comparisons with immunized mare titers were only made between all 7 control samples collected on the day of foaling (D0 post-foaling (PF)) with 12 vaccinated samples collected pre-immunization, on day 21 prior to the booster dose, and on D0 PF (S1 Fig). When compared with IgG titers to PNAG in non-immune controls obtained on D0 PF, immunization of mares against PNAG gave rise to significant (P < 0.05) increases in total serum IgG titers as well as increases in the titers of equine IgG subisotypes IgG_1_, IgG_3/5_, and IgG_4/7_ (S1 Fig) on day 21 after a single immunization, and on D0 PF after the booster dose. Similarly, total IgG and IgG subisotype titers were significantly higher in the colostrum obtained on the day of foaling from vaccinated mares compared with controls (S2 Fig). Notably, non-immunized mares had antibody titers to PNAG, representative of the natural response to this antigen commonly seen in normal animal and human sera.

**Fig 1:**
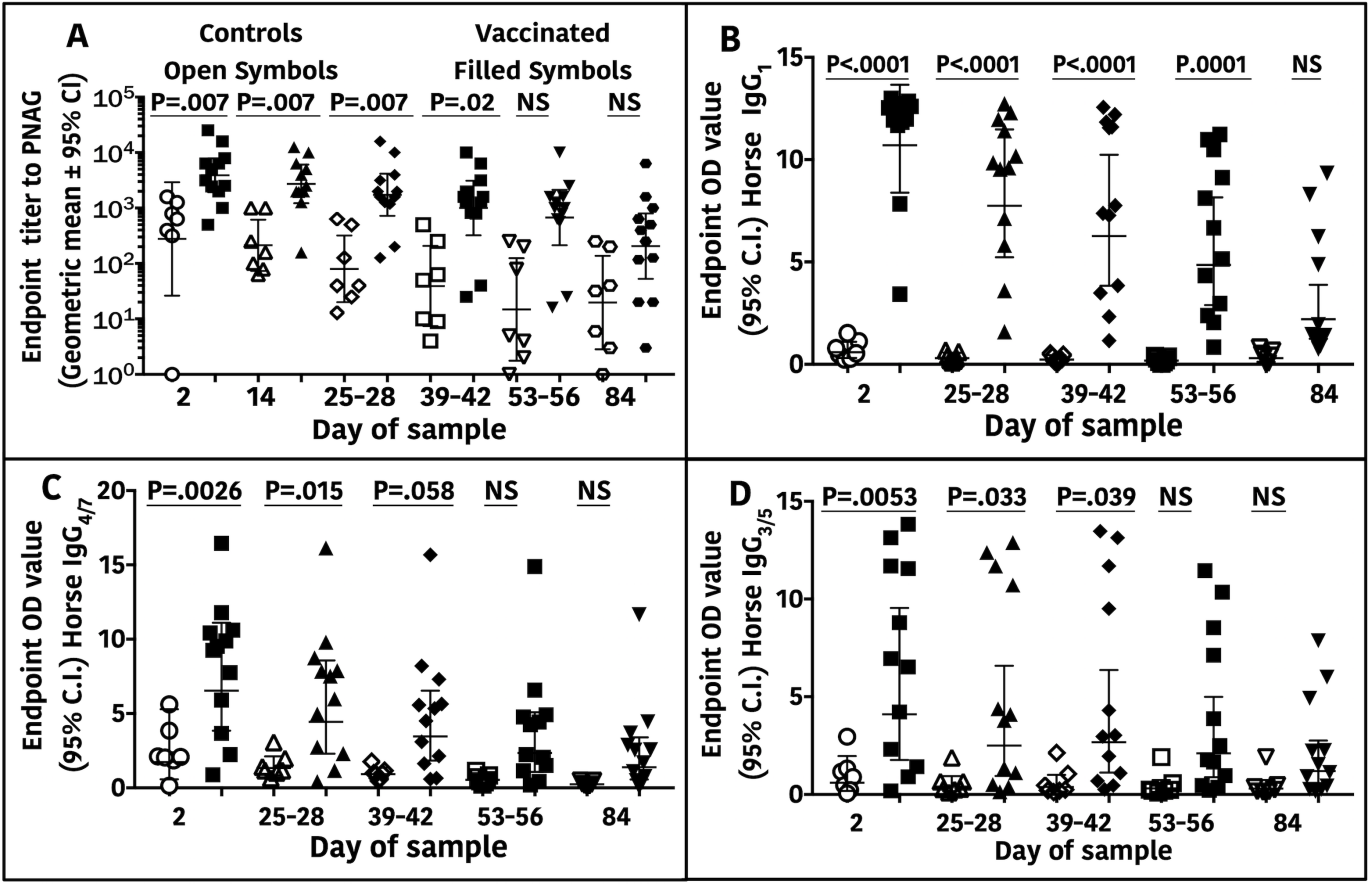
Total IgG and IgG subisotype antibody titers to PNAG in sera of horse foals. Endpoint serum titers (N = 7 controls, 12 vaccinated) of IgG or IgG subisotypes are plotted by vaccine group as a function of age in days. **A**: IgG antibody end-point titers to PNAG were significantly higher in an age-dependent matter between foals from mares that were vaccinated (filled symbols n = 12) compared with titers in sera of foals from unvaccinated, control mares (open symbols n = 7) through Days 39–42 of life. **B-D**: Concentrations of IgG_1,_ IgG_4/7_, and IgG_3/5_ to PNAG were significantly higher in foals in the vaccinated group than the unvaccinated, control group through the day indicated on the figure. Statistical comparisons made using linear mixed-effects modeling with individual foal as a random effect; NS = not significant.

Successful oral delivery of antibody to the blood of foals born to vaccinated mares (hereafter termed vaccinated foals) was shown by the significantly higher titers of serum IgG to PNAG compared with foals from control mares at ages 2, 28, and 56 days, but not 84 days (Fig 1A). Foal serum concentrations of subisotypes IgG_1_, IgG_3/5_, and IgG_4/7_ to PNAG were significantly higher at 2, 28, and 42 days of age in the vaccinated group compared with the control group, and subisotype IgG_1_ titers remained significantly higher through age 56 days (Fig 1B–1D). The pattern in vaccinated foals of decreasing titers to PNAG with increasing age was consistent with the decay of maternally-transferred immunoglobulins.

### Orally obtained colostral antibody to PNAG protects foals against intrabronchial infection with *R*. *equi*

**Fig 2:**
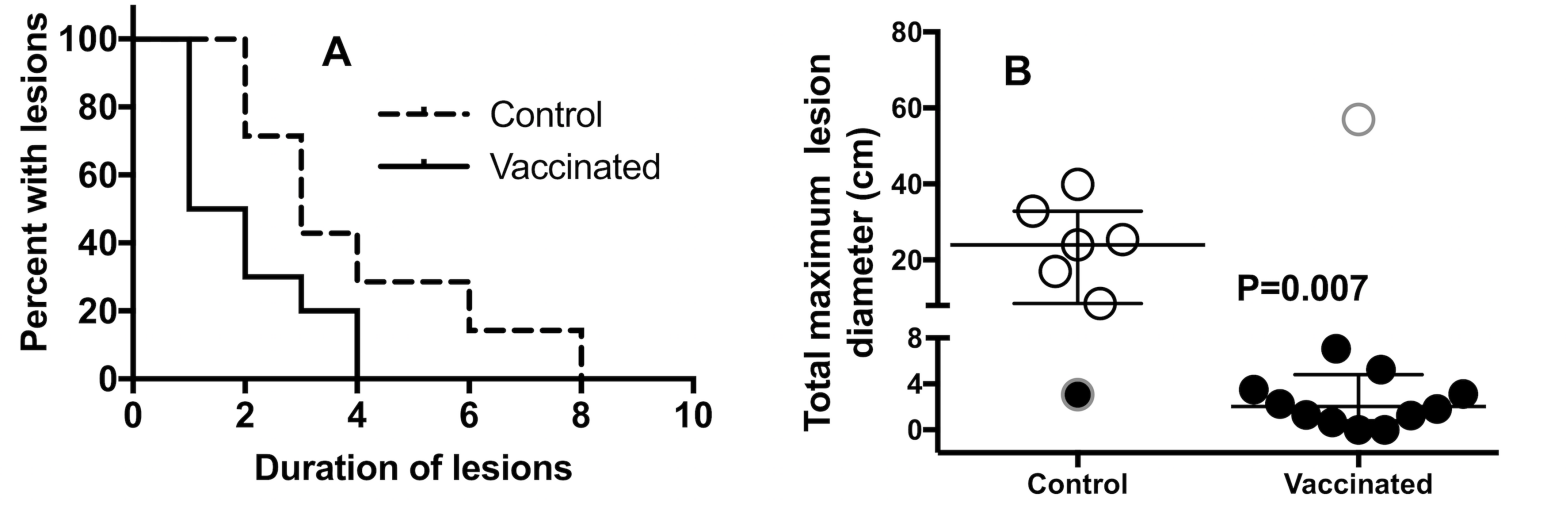
Comparison of induction and regression of ultrasonographic lesions in foals from vaccinated or unvaccinated mares following *R*. *equi* challenge. **A:** Kaplan-Meier survival plot comparing duration of detectable ultrasonographic lesions as evidence of pulmonary abscessation. Duration of pulmonary lesions identified by ultrasound was significantly (P = 0.008; Log-rank test) shorter for foals of vaccinated mares (solid line) versus those of foals from control mares (hatched line). **B:** Cumulative sum of maximum diameters of thoracic ultrasonography lesions (N = 7 Controls, 12 Vaccinated). The sums of the cumulative maximum diameters were significantly (P = 0.007; Wilcoxon rank-sum test) lower for foals from vaccinated mares (n = 12) than for unvaccinated control mares (n = 7). Open circles indicate foals diagnosed with pneumonia, filled circles indicate foals that did not develop pneumonia. Symbols with outer gray rings indicate the unvaccinated foal that did not get pneumonia and the vaccinated foal that did develop pneumonia.

Protection studies were undertaken using a randomized, controlled, blinded experimental trial design. At days 25–28 of life, foals in the study were challenged with ~10^6^ CFU of live *R*. *equi* contained in 40 ml of vehicle, with half of the challenge delivered to each lung by intrabronchial dosing with 20 ml. Foals were followed for development of clinical *R*. *equi* pneumonia (Table 1) for 8 weeks. The proportion of vaccinated foals that developed *R*. *equi* pneumonia (8%; 1/12) was significantly (P = 0.0017; Fisher’s exact test) less than that of unvaccinated control foals (86%; 6/7), representing a relative risk reduction or protected fraction of 84% (95% C.I. 42% to 97%, Koopman asymptotic score analysis [27]). The duration of clinical signs indicative of *R*. *equi* pneumonia was significantly (P ≤ 0.027, Wilcoxon rank-sum tests) longer for foals from control than vaccinated mares (Table 2). Thoracic ultrasonographic examination is the standard clinical technique for monitoring areas of pulmonary abscessation or consolidation attributed to *R*. *equi* infection. The severity and duration of ultrasonographic lesions were significantly greater in foals born to controls than vaccinated mares (Fig 2). Vaccinated foals that were protected against pneumonia had less severe clinical signs and smaller and fewer ultrasonographic lesions compared with control foals. Thus, maternal vaccination against PNAG demonstrated successful protection against clinical *R*. *equi* pneumonia, a disease for which there is no current vaccine [11], using a randomized, blinded experimental challenge model.

**Table 1:** Case definition for diagnosis of *R*. *equi* clinical pneumonia.

**Cases had all of the following clinical signs within 3 weeks of challenge:**
Coughing
Depressed attitude (subjective evidence of increased recumbency, lethargy, reluctance to rise)
Fever > 103.5°F
Tachypnea (respiratory rate > 60 breaths/min)
Increased respiratory effort (abdominal lift, flaring nostrils)
Sonographic evidence of pulmonary abscessation or consolidation
Cytologic evidence of septic pneumonia from tracheal aspirate
Positive culture for *R. equi* from tracheal aspirate
**Lack of diagnosis with clinical *R*. *equi* pneumonia was based on lack of any of the following clinical signs within 8 weeks post-challenge:**
Coughing
Depressed attitude (subjective evidence of increased recumbency, lethargy, reluctance to rise)
Fever > 103.5°F
Tachypnea (respiratory rate ≥ 60 breaths/min)
Increased respiratory effort (abdominal lift, flaring nostrils)

**Table 2:** Duration of clinical signs in foals from vaccinated or unvaccinated mares.

Variable	Mare Unvaccinated (n = 7)	Mare Vaccinated (n = 12)	P value^a^
Days meeting case definition (range)	10^b^ (0 to 26)	0 (0 to 41)	0.0046
Days from first to last day meeting case definition (range)	20 (0 to 32)	0 (0 to 48)	0.0046
Days temperature > 103.0°F (range)	8 (1 to 32)	2 (0 to 12)	0.0263
Days temperature > 103.0°F and coughing (range)	4 (0 to 8)	0 (0 to 13)	0.0220

^a^P values derived using the Wilcoxon rank-sum test.

^b^Median (range)

### Passive infusion with hyperimmune plasma to PNAG protects foals against *R*. *equi* pneumonia

To substantiate that vaccination-mediated protection was attributable to antibody to PNAG, hyperimmune plasma was prepared from the blood of 5GlcNH_2_-TT-immunized adult horses and 2 L (approximately 40 ml/kg) infused into 5 foals at 18–24 hours of age. Four controls were transfused at the same age with 2 L of standard commercial horse plasma. Titers of control and hyperimmune plasma IgG subisotypes and IgA antibody to PNAG and OPK activity against *R*. *equi* (S3 Fig) documented significantly higher titers of functional antibody to PNAG in the plasma from vaccinated donors and in foals transfused with the plasma from vaccinated donors compared to foals transfused with standard plasma. After challenge with *R*. *equi* as described above, there was a significant reduction in clinical signs in the foals receiving PNAG-hyperimmune plasma, compared to controls, except for the duration of ultrasound lesions (Table 3). None of the 5 foals receiving PNAG-hyperimmune plasma were diagnosed with *R*. *equi* pneumonia, whereas 4 of 4 recipients of normal plasma had a diagnosis of clinical pneumonia for at least 1 day (P = 0.0079, Fisher’s exact test; relative risk reduction or protected fraction 100%, 95% C.I. 51%-100%, Koopman asymptotic score [27]).

**Table 3:** Duration of clinical signs in foals infused with control or PNAG-hyperimmune plasma.

Variable	Standard plasma (n = 4)	PNAG plasma (n = 5)	P value^a^
Days meeting case definition (range)	10.5^b^ (1 to 14)	0 (0 for all)	0.0108
Days from first to last day meeting case definition (range)	11.5 (1 to 14)	0 (0 for all)	0.0104
Days temperature > 103.0°F (range)	11.5 (1 to 15)	2 (0 to 4)	0.0811
Days temperature > 103.0°F and coughing (range) Duration of ultrasound lesions in weeks (range) Maximum of TMD^c^ (cm) Sum of TMDs (cm)	9.5 (0 to 11) 2 (1 to 4) 10.1 (4.0 to 29.8) 13.2 (4.0 to 50.5)	0 (0 to 3) 0 (0 to 3) 0 (0 to 3.4) 0 (0 to 7.9)	0.0072 0.2029 0.0179 0.0342

^a^P values derived using the Mann-Whitney test.

^b^Mean (range)

^c^TMD = total (sum) of maximum diameters of lesions observed on a given day

### *R*. *equi* expression of PNAG *in vitro* and *in vivo*

Using immunofluorescence microscopy, we demonstrated that 100% of 14 virulent strains of *R*. *equi* tested express PNAG (S4 Fig). Moreover, we found that PNAG was expressed in the lungs of foals naturally infected with *R*. *equi* (S5A Fig), similar to our prior demonstration of PNAG expression in the lung of a human infected with Mtb [13]. PNAG was detected within apparent vacuoles inside *R*. *equi*-infected horse macrophages *in vivo* (S5B Fig).

### PNAG vaccine-induced opsonic antibodies mediate killing of both extracellular and intracellular *R*. *equi*

**Fig 3:**
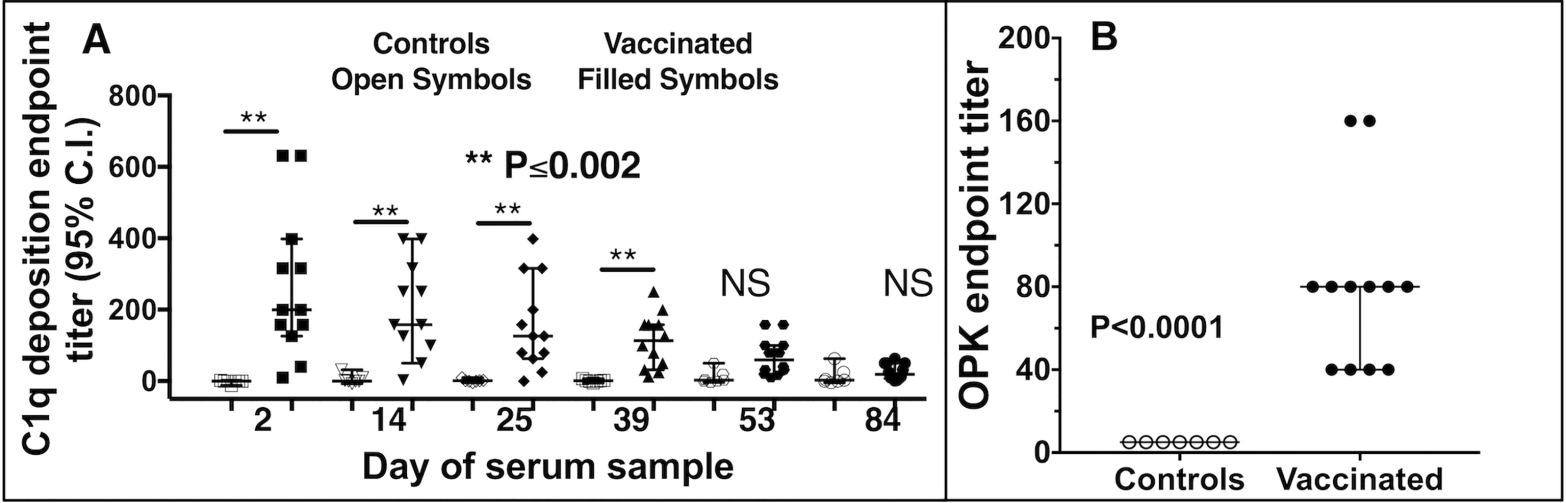
Functional activity of antibody in foal sera on day of challenge with *R*. *equi*. **A**: Serum endpoint titer (N = 7 Controls, 12 Vaccinated) of deposition of equine C1q onto purified PNAG. P values determined by non-parametric ANOVA and pairwise comparisons by Dunn’s procedure. NS, not significant. **B**: Serum endpoint titer (reciprocal of serum dilution achieving killing ≥ 30% of input bacteria) for opsonic killing of *R*. *equi* in suspension along with horse complement and human PMN. Values indicate individual titer in foal sera on day of challenge with *R*. *equi*, black bars the group median and error bars the 95% C.I. (upper 95% C.I for vaccinated foals same as median). P value by Wilcoxon rank-sum test.

Testing of the functional activity of the antibodies induced in the pregnant mares and in foal sera on the day of challenge demonstrated the antibodies could fix equine complement component C1q onto the PNAG antigen (Fig 3A). Notably, the natural antibody to PNAG in sera of non-vaccinated, control mares and their foals did not deposit C1q onto the PNAG antigen, consistent with prior findings that natural antibodies are immunologically inert in these assays [15, 16, 28]. Sera from vaccinated foals on the day of *R*. *equi* infection mediated high levels of opsonic killing of extracellular *R*. *equi* whereas control foals with only natural maternal antibody to PNAG had no killing activity (Fig 3B), again demonstrating the lack of functional activity of these natural antibodies to PNAG.

As some of the vaccinated foals developed small subclinical lung lesions that resolved rapidly (Table 1, Fig 2) it appeared the bolus challenge did lead to some uptake of *R*. *equi* by alveolar macrophages but without development of detectable clinical signs of disease. This observation suggested that antibody to PNAG led to resolution of these lesions and prevented the emergence of clinical disease. Based on the finding that *R*. *equi*-infected foal lung cells expressed PNAG *in vivo* (S5 Fig), we determined if macrophages infected with *R*. *equi in vitro* similarly expressed PNAG, and also determined if this antigen was on the infected cell surface, intracellular, or both. We infected cultured human monocyte-derived macrophages (MDM) for 30 min with live *R*. *equi* then cultured them overnight in antibiotics to prevent extracellular bacterial survival. To detect PNAG on the infected cell surface we used the human IgG1 MAb to PNAG (MAb F598) conjugated to the green fluorophore Alexa Fluor 488. To detect intracellular PNAG, we next permeabilized the cells with ice-cold methanol and added either unlabeled MAb F598 or control MAb, F429 [29] followed by donkey anti-human IgG conjugated to Alex Fluor 555 (red color). These experiments showed there was no binding of the MAb to uninfected cells (S6A Fig) nor binding of the control MAb to infected cells (S6B Fig). However, we found strong expression of PNAG both on the infected MDM surface and within infected cells (S6C Fig). Similarly, using a GFP-labeled Mtb strain (S6D and S6E Fig) and a GFP-labeled strain of *Listeria monocytogenes* (S6F Fig) we also visualized intense surface expression of PNAG on infected human MDMs in culture, even when the bacterial burden in the infected cell was apparently low. Importantly, within infected cultures, only cells with internalized bacteria had PNAG on their surface (S6G Fig), indicating the antigen originated from the intracellular bacteria. Thus, cells in infected cultures that did not ingest bacteria did not obtain PNAG from shed antigen or lysed infected cells. This finding is consistent with published reports of intracellular bacterial release of surface vesicles that are transported among different compartments of an infected host cell [30].

**Fig 4:**
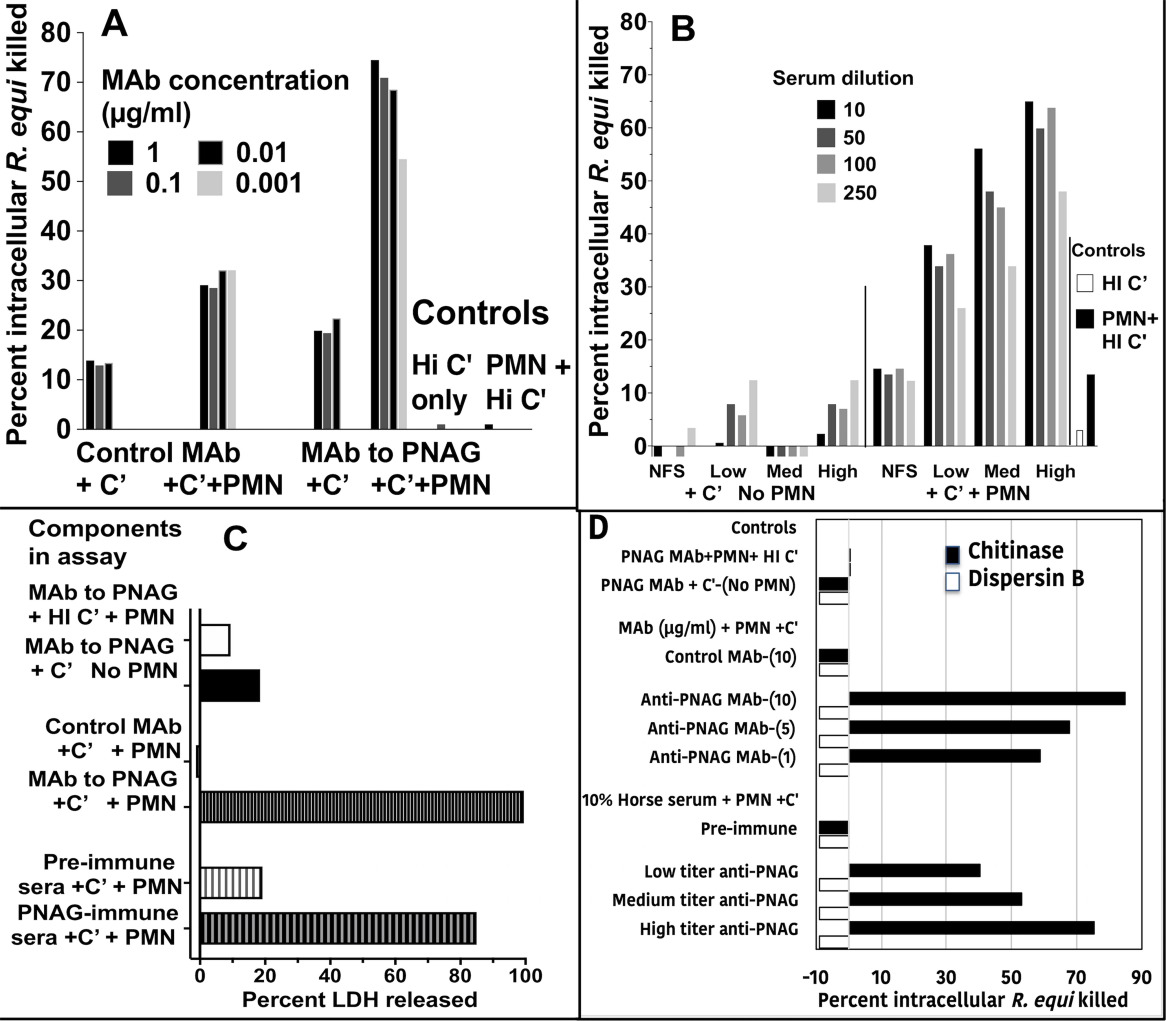
Opsonic killing of intracellular *R*. *equi*. **A:** Maximal killing of intracellular *R*. *equi* mediated by MAb to PNAG requires both complement (C’) and PMN (C’+PMN). Background killing <5% is achieved with heat-inactivated C’ (HI C’) or PMN + HI C’. **B:** Pre-immune, normal foal sera (NFS) or representative immune foal sera with low, medium (Med) or high titers to PNAG obtained on the day of challenge with *R*. *equi* mediate killing of intracellular *R*. *equi* along with C’ and PMN. **C:** Measurement of percent cytotoxicity by LDH release shows MAb to PNAG or PNAG-immune sera plus C’ and PMN mediate lysis of infected cells. **D:** Opsonic killing of intracellular *R*. *equi* requires recognition of cell surface PNAG. Treatment of infected macrophage cultures with dispersin B to digest surface PNAG eliminates killing whereas treatment with the control enzyme, chitinase, has no effect on opsonic killing. Bars represent means of 4–6 technical replicates. Depicted data are representative of 2–3 independent experiments. Bars showing <0% kill represent data wherein the cfu counts were greater than the control of PNAG MAb + PMN + HI C’.

Next, we examined if the surface PNAG on infected cells provided the antigenic target needed by antibody to both identify infected cells and, along with complement and PMN, lyse the cells, release the intracellular microbes, and kill them by classic opsonic killing. Human MDM cultures were established *in vitro*, infected for 30 min with live *R*. *equi*, and then cells were washed and incubated for 24 h in the presence of 100 μg gentamicin/ml to kill extracellular bacteria and allow for intracellular bacterial growth. Then, various combinations of the human IgG_1_ MAb to PNAG or the control MAb F429 along with human complement and human PMN were added to the cultures, and viable *R*. *equi* determined after 90 min. While a low level of killing (≤30%) of intracellular *R*. *equi* was obtained with PMN and complement in the presence of the control MAb, there was a high level of killing of the intracellular *R*. *equi* when the full compendium of immune effectors encompassing MAb to PNAG, complement, and PMN were present (Fig 4A). Similarly, testing of sera from vaccinated foals on the day of challenge, representing animals with a low, medium, or high titer of IgG to PNAG, showed they also mediated titer-dependent killing of intracellular *R*. *equi* (Fig 4B). Measurement of the release of lactate dehydrogenase as an indicator of lysis of the macrophages showed that the combination of antibody to PNAG, complement, and PMN mediated lysis of the infected human cells (Fig 4C), presumably releasing the intracellular bacteria for further opsonic killing.

PNAG can be digested with the enzyme dispersin B that specifically recognizes the β-1→6-linked *N*-acetyl glucosamine residues [31, 32] but is unaffected by chitinase, which degrades the β-1→4-linked *N*-acetyl glucosamines in chitin. Thus, we treated human macrophages infected for 24 h with *R*. *equi* with either dispersin B or chitinase to determine if the presence of surface PNAG was critical for killing of intracellular bacteria. Dispersin B treatment markedly reduced the presence of PNAG on the infected cell surface (S7 Fig) as well as killing of intracellular *R*. *equi* by antibody, complement, and PMN (Fig 4D). Chitinase treatment had no effect on PNAG expression (S7 Fig) or killing, indicating a critical role for PNAG intercalated into the macrophage membrane for antibody-mediated killing of intracellular *R*. *equi*.

### Antibody to PNAG mediates intracellular killing of other intracellular pathogens

**Fig 5:**
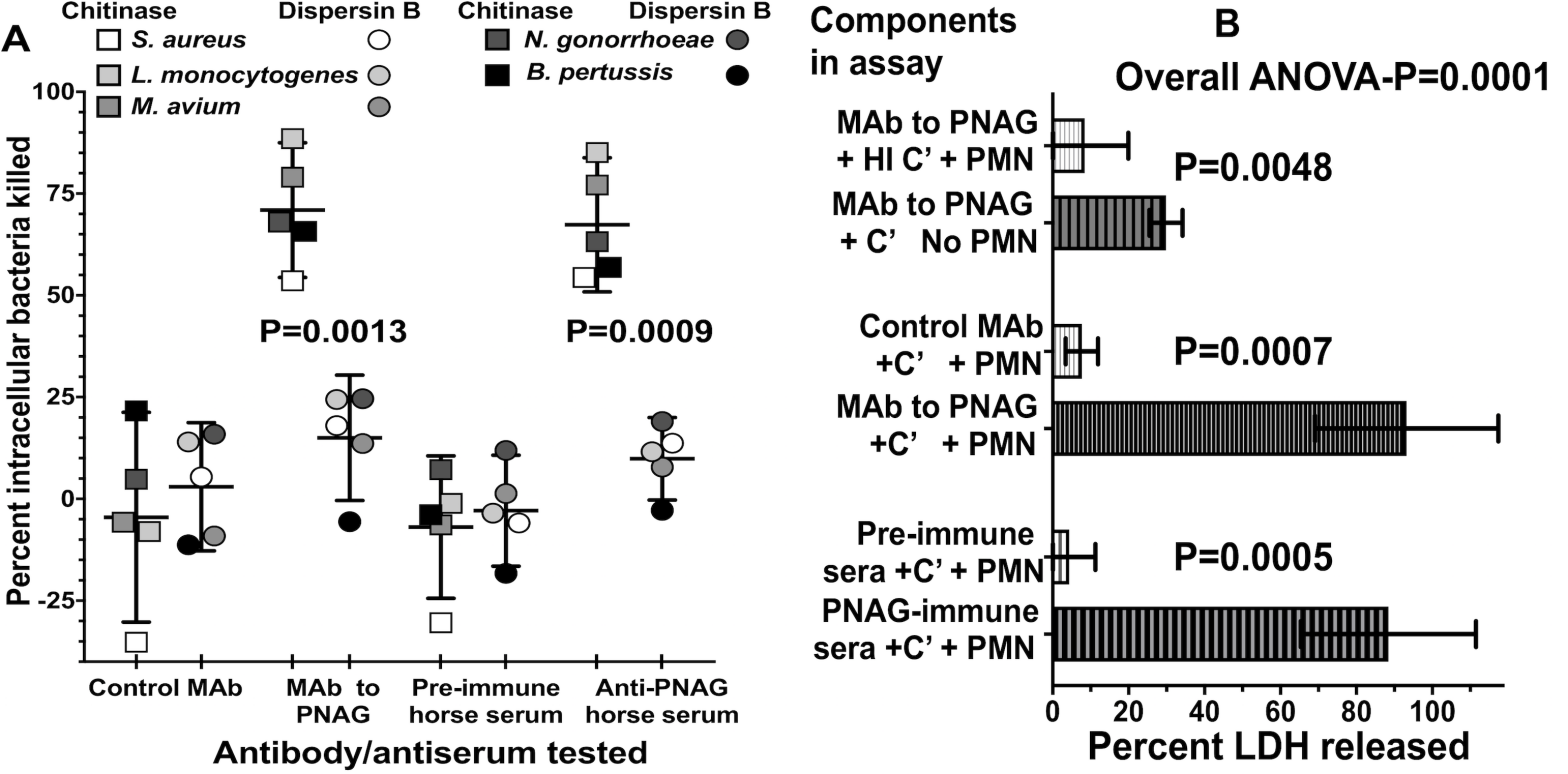
Opsonic killing of multiple intracellular pathogens by antibody to PNAG, complement (C’) and PMN depends on infected-cell surface expression of PNAG and is associated with release of LDH. **A:** Killing of 5 different intracellular bacterial pathogens by monoclonal or polyclonal antibody (10% concentration) to PNAG plus PMN and C’ was markedly reduced following treatment of infected cells with Dispersin B (circles) to digest surface PNAG compared to treatment with the control enzyme, Chitinase (squares). Symbols represent indicated bacterial target strain. Horizontal bars represent means, and error bars show the 95% C.I. Symbols showing <0% kill represent data wherein the cfu counts were greater than the control of PNAG MAb + PMN + HI C’. P values: paired t-tests comparing percent intracellular bacteria killed with each antibody/antiserum tested after Chitinase or Dispersin B treatment. **B:** Opsonic killing is associated with maximal LDH release from infected cells in the presence of antibody to PNAG, C’ and PMN. Bars represent means from 5 different intracellular pathogens, error bars the 95% C.I., overall ANOVA P value by one-way repeated measures ANOVA, pair wise comparisons determined by two-stage linear step-up procedure of Benjamini, Krieger and Yekutieli.

To show that antibody to PNAG, complement, and PMN represent a general mechanism for killing of disparate intracellular pathogenic bacteria that express PNAG, we used the above-described system of infected human macrophages to test killing of *Mycobacterium avium*, *Staphylococcus aureus*, *Neisseria gonorrhoeae*, *Listeria monocytogenes* and *Bordetella pertussis* by the human MAb to PNAG or horse serum from a foal protected from *R*. *equi* pneumonia. Human MDM infected with these organisms expressed PNAG on the surface that was not detectable after treatment with dispersin B (S7 Fig). When present intracellularly, all of these organisms were killed in the presence of MAb to PNAG or anti-PNAG immune horse serum, complement, and PMN following treatment of the infected cells with the control enzyme, chitinase, but killing was markedly reduced in infected cells treated with dispersin B (Figs 5A and S8). As with *R*. *equi*, maximal lysis of infected cells occurred when antibody to PNAG plus complement and PMN were present (Fig 5B and S9 Fig), although when analyzing data from all 5 of these experiments combined there was a modest but significant release of LDH with antibody to PNAG and complement alone (Fig 5B and S9 Fig).

### Maternal PNAG vaccination and antibody transfer to foals enhances *in vitro* cell-mediated immune responses against *R*. *equi*

**Fig 6:**
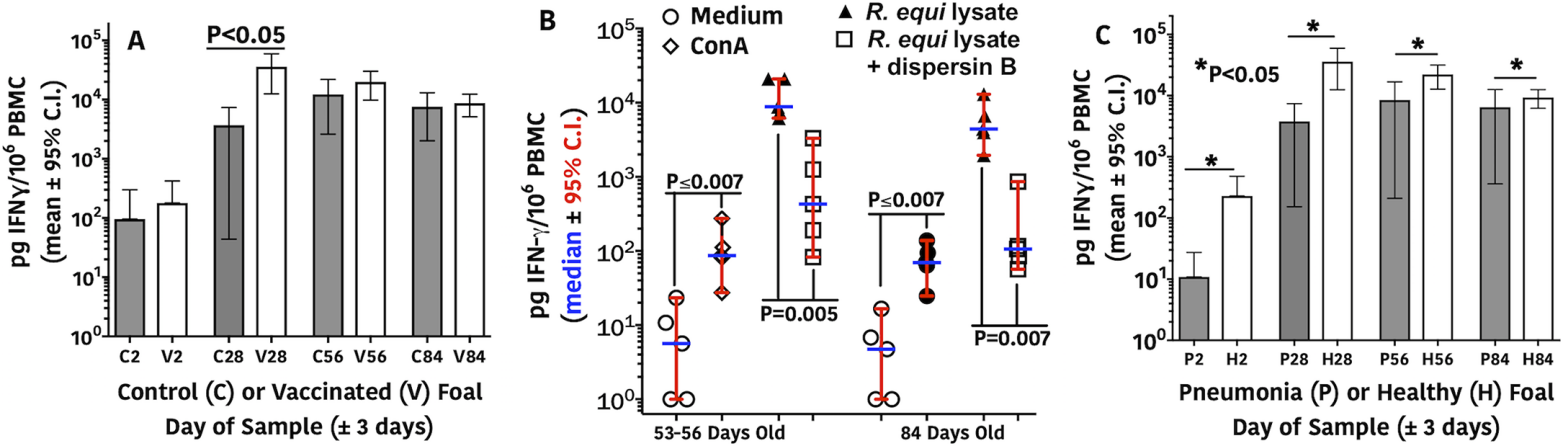
Cell-mediated immune responses of foal PBMCs. **A:** Foals (N = 7 controls, 12 vaccinated) from vaccinated mares (V) had significantly (P <0.05; linear-mixed effects modeling) higher concentrations of IFN-γ produced at 28 days of age (prior to challenge) than control (C) foals in response to stimulation by a lysate of *R*. *equi*. IFN-γ production at 2 days of age was significantly (P <0.05; linear mixed-effects modeling) lower than those at all other days for both the control and vaccine groups (P values not shown on graph). **B:** IFN-γ production from PBMC from 5 vaccinated foals at 56 and 84 days of age following intrabronchial infection with virulent *R*. *equi*. Stimuli included media only (negative control), Concanavilin A (ConA; positive control), lysate of virulent *R*. *equi* strain used to infect the foals (*R*. *equi* lysate); and the same lysate treated with dispersin B to digest PNAG. All 3 stimulated groups were significantly different from the medium control at both day 56 and 84 (Overall ANOVA for repeated measures (P < 0.0001); P ≤ 0.0070 for all pairwise comparisons to media only and for pairwise comparison for *R*. *equi* lysate vs. lysate plus dispersin B (indicated on top of graph), Holm-Sidak's multiple comparisons test. **C:** Foals (N = 7 controls, 12 vaccinated) that developed pneumonia (P) had significantly (P < 0.05; linear-mixed effects modeling) lower concentrations of IFN-γ expression at each day relative to foals that remained healthy (H).

Cell-mediated immune (CMI) responses in vaccinated and unvaccinated, control foals were assessed by detecting production of IFN-γ from peripheral blood mononuclear cells (PBMC) stimulated with a lysate of virulent *R*. *equi*. IFN-γ production at 2 days of age was significantly (P < 0.05; linear mixed-effects modeling) lower than levels at all other days for both the control and vaccinated groups (Fig 6A, P value not on graph). There was no difference in IFN-γ production between vaccinated and control foals at day 2 of age. Vaccinated foals had significantly higher (~10-fold) production of IFN-γ in response to *R*. *equi* stimulation (Fig 6A) from cells obtained just prior to challenge on days 25–28 of life compared to unvaccinated controls. By 56 days of age, and 4 weeks post *R*. *equi* infection, the controls likely made a CMI response to the lysate antigens as they were infected at day 25–28 of life, accounting for the lack of differences between vaccinates and controls in PBMC IFN-γ production at day 56.

To substantiate the specificity of this CMI reaction from the PBMC of vaccinated foals, we demonstrated that stimulation of their PBMC with an *R*. *equi* lysate treated with the enzyme dispersin B diminished IFN-γ responses by ~90% (Fig 6B). We did not test PBMC from control foals for specificity of their responses to PNAG. We also made a *post hoc* comparison of CMI responses between foals that remained healthy and foals that developed pneumonia. In this analysis (Fig 6C), foals that remained healthy (11 vaccinates and 1 control) had significantly (P < 0.05; linear mixed-effects modeling) higher CMI responses at all ages, including age 2 days, than foals that became ill (1 vaccinate and 6 controls), suggesting that both innate and acquired cellular immunity contribute to resistance to *R*. *equi* pneumonia. Overall, it appears the maternally derived antibody to PNAG sensitizes foal PBMC to recognize the PNAG antigen and release IFN-γ, which is a known effector of immunity to intracellular pathogens.

## Discussion

In this study we tested the hypotheses that antibody to the conserved surface microbial polysaccharide, PNAG, could mediate protection against a significant intracellular pathogen of horse foals, *R*. *equi*. Overall we supported this hypothesis by showing maternal immunization against the deacetylated glycoform PNAG induced antibodies that protected ~4-week-old foals from challenge with live, virulent *R*. *equi*. Mechanistically we found that vaccine-induced antibody to PNAG deposited complement component C1q onto the purified PNAG antigen, mediated opsonic killing of both extracellular and intracellular *R*. *equi*, and sensitized PBMC from vaccinated foals to release IFN-γ in response to PNAG. It appears that this spectrum of antibody activity induced by the 5GlcNH_2_-TT vaccine were all critical to the protective efficacy observed.

While immunization-challenge studies such as those performed here are often correlative with protective efficacy against infection and disease, such studies can have limitations in their ability to predict efficacy in the natural setting. Bolus challenges provide an acute insult and immunologic stimulus that mobilizes immune effectors and clears infectious organisms, whereas in a field setting, such as natural acquisition of *R*. *equi* by foals, infection likely occurs early in life with onset of disease signs taking several weeks to months to develop [5, 6]. Thus, it cannot be predicted with certainty that the protective efficacy of antibody to PNAG manifest in the setting of acute, bolus challenge will also be effective when a lower infectious inoculum and more insidious course of disease develops. In the context of acute challenge, we noted that many of the protected, vaccinated foals developed small lung lesions after challenge that rapidly resolved and no disease signs were seen. Finding such lesions by routine ultrasound examination of foals that occurs on farms [33] might instigate treatment of subclinical pneumonia if equine veterinarians are either unwilling to monitor foals until clinical signs appear or unconvinced that disease would not ensue in vaccinated foals. This approach could obviate the benefit of vaccination.

The protection studies described here for *R*. *equi* disease in foals has led to the implementation of a human trial evaluating the impact of infusion of the fully human IgG_1_ MAb to PNAG on latent and new onset TB. The MAb has been successfully tested for safety, pharmacokinetic, and pharmacodynamic properties in a human phase I test [34]. The trial in TB patients began in September 2017 (South African Clinical Trials Register: http://www.sanctr.gov.za/SAClinicalbrnbspTrials/tabid/169/Default.aspx, then link to respiratory tract then link to tuberculosis, pulmonary; and TASK Applied Sciences Clinical Trials, AP-TB-201-16 (ALOPEXX): https://task.org.za/clinical-trials/). The MAb was chosen for initial evaluation to avoid issues of variable immunogenicity that might arise if a vaccine were tried in a TB-infected population, and to have a greater margin of safety in case of untoward effects of immunity to PNAG in the human setting. It is expected the half-life of the MAb will lead to its reduction to pre-infusion levels over 9 to 15 months whereas this might not be the case following vaccination. A successful effect of the MAb on treatment or disease course in TB will lead to an evaluation of immunogenicity and efficacy of a PNAG targeting vaccine in this patient population. The vaccine used here in horse mares was part of a batch of material produced for human phase 1 safety and immunogenicity testing (ClinicalTrials.gov Identifier: NCT02853617), wherein early results indicate that among a small number of vaccinates there were no serious adverse events and high titers of functional antibody elicited in 7 of 8 volunteers given either 75 μg or 150 μg doses twice 28 days apart. As part of the safety evaluation, vaccinates kept daily logs of health status, which focused on potential signs or symptoms of disease resulting from disruption of normal microbial flora. This is not only a well-known consequence of antibiotic treatments [35], but also can occur from many licensed and experimental drugs [36] across all major drug classes. No adverse events attributable to microflora changes were reported. In addition, we have previously published an extensive analysis of the low potential of antibody to PNAG to impact the normal microbial flora [13].

Numerous investigators have studied how antibodies can mediate protection against intracellular bacterial pathogens [37–39], although specific mechanisms of immunity are not well defined. The *in vitro* results we derived indicated that a cell infected with a PNAG-producing pathogen has prominent surface display of this antigen that serves as a target for antibody, complement and PMN to lyse the infected cell and release the intracellular organisms for subsequent opsonic killing. Likely other bacterial antigens are displayed on the infected host cell as well, and thus this system could be used to evaluate the protective efficacy and mechanism of killing by antibodies to other antigens produced by intracellular organisms. Although we have not investigated the basis for the appearance of PNAG in the plasma membrane of infected host cells, we suspect that microbial extracellular vesicles, known to be released by many microbes [40], are a likely source of the plasma membrane antigen due to trafficking from infected cellular compartments [30].

A notable component of the immune response in the foals associated with the protective efficacy of the maternally derived antibody was the release of IFN-γ from PBMC in response to a *R*. *equi* cell lysate. The response to the lysate significantly dropped after treatment of the lysate with the PNAG-degrading enzyme dispersin B, indicating that an antibody-dependent cellular response to PNAG underlay the IFN-γ response. As this cytokine is well known to be an important component of resistance to intracellular pathogens [41], it was notable that the maternal immunization strategy led to an antibody-dependent IFN-γ response from the PBMC of the vaccinated foals. After challenge with *R*. *equi*, the control foals also developed an IFNγ-PBMC response. It also appears that the reliance on traditional T-cell effectors recognizing MHC-restricted microbial antigens to provide components of cellular immunity can potentially be achieved with an antibody-dependent mechanism of cellular responses, further emphasizing how antibody can provide immunity to intracellular pathogens.

This study addressed many important issues related to vaccine development, including the utility of maternal immunization to provide protection against an intracellular pathogen via colostrum to immunologically immature offspring, the efficacy and mechanism of action of antibody to PNAG in protective efficacy, and identification of a role for antibody-dependent IFN-γ release in the response to immunization that likely contributed to full immunity to challenge. The success of immunization in protecting against *R*. *equi* challenge in foals targeting the broadly synthesized PNAG antigen raises the possibility that this single vaccine could engender protection against many microbial pathogens. While the potential to protect against multiple microbial targets is encouraging, the findings do raise issues as to whether antibody to PNAG will be protective against many microbes or potentially manifest some toxicities or unanticipated enhancements of infection caused by some organisms. Thus, continued monitoring and collection of safety data among animals and humans vaccinated against PNAG is paramount until the safety profile of antibody to PNAG becomes firmly established. Overall, the protective efficacy study in foals against *R*. *equi* has initiated the pathway to development of PNAG as a vaccine for significant human and animal pathogens, and barring unacceptable toxicity, the ability to raise protective antibodies to PNAG with the 5GlcNH_2_-TT conjugate vaccine portends effective vaccination against a very broad range of microbial pathogens.

## Materials and methods

### Experimental design

The objective of the research was to test the ability of maternal vaccination of horse mares with a conjugate vaccine targeting the PNAG antigen to deliver, via colostral transfer, antibody to their offspring that would prevent disease due to intrabronchial *R*. *equi* challenge at ~4 weeks of life. A confirmatory study using passive infusion of immune or control horse plasma to foals in the first 24 hours of life was also undertaken. The main research subjects were the foals; the secondary subjects were the mares and their immune responses. The experimental design was a randomized, controlled, experimental immunization-challenge trial in horses, with pregnant mares and their foals randomly assigned to the vaccine or control group. Group assignment was made using a randomized, block design for each year. Data were obtained and processed randomly then pooled after unblinding for analysis. Investigators with the responsibility for clinical diagnosis were blinded to the immune status of the foals. An unblinded investigator monitored the data collected to ascertain lack of efficacy and stopping of the infections if 5 or more vaccinated foals developed pneumonia. A similar design was used for the transfusion/passive immunization study, except for the stopping rule.

### Samples size determination

The sample size for the foal protection study was based on prior experience with this model [5, 10, 42] indicating a dose of 10^6^ CFU of *R*. *equi* delivered in half-portions to the left and right lungs via intrabronchial instillation would cause disease in ~85% of foals. Thus, a control group of 7 foals, anticipating 6 illnesses, and a vaccinated group of 12 foals, would have the ability to detect a significant effect at a P value of <0.05 if 75% of vaccinated foals were disease-free using a Fisher’s exact test, based on the use of the hypergeometric distribution that underlies the experimental design wherein there is no replacement of a subject into the potential experimental outcomes once it is diagnosed as ill.

All clinical and immunological data to be collected were defined prior to the trial in mares and foals, and no outliers were excluded from the analysis. The primary endpoint was development of clinical *R*. *equi* pneumonia as defined under Clinical Monitoring below. Experiments were performed over 3 foaling seasons: 2015 and 2016 for the active immunization of pregnant mares, with results from the 2 years of study combined, and 2017 for the passive infusion study.

### Ethics statement

All procedures for this study were reviewed and approved by the Texas A&M Institutional Animal Care and Use Committee (protocol number AUP# IACUC 2014–0374 and IACUC 2016–0233) and the University Institutional Biosafety Committee (permit number IBC2014-112). The foals used in this study were university-owned, and permission for their use was provided in compliance with the Institutional Animal Care and Use Committee procedures. No foals died or were euthanized as a result of this study.

### Vaccine

Mares in the vaccine group received 125 μg (during 2015) or 200 μg (2016) of synthetic pentamers of β-1→6-linked glucosamine conjugated to tetanus toxoid (ratio of oligosaccharide to protein 35–39:1; AV0328, Alopexx Enterprises, LLC, Concord, MA) diluted to 900 μl in sterile medical grade physiological (*i*.*e*., 0.9% NaCl) saline solution (PSS) combined with 100 μl of Specol (Stimune Immunogenic Adjuvant, Prionics, Lelystad, Netherlands, now part of Thermo-Fischer Scientific), a water-in-oil adjuvant. The rationale for increasing the dose in 2016 was that some vaccinated mares had relatively low titers, although all foals of vaccinated mares born in 2015 were protected. Mares in the unvaccinated group were sham injected with an equivalent volume (1 ml) of sterile PSS. All pregnant mares were vaccinated or sham vaccinated 6 and 3 weeks prior to their estimated due dates. For the transfusion of hyperimmune plasma, adult horses (not pregnant) were immunized as above, blood obtained, and hyperimmune plasma produced from the blood by the standard commercial techniques used by Mg Biologics, Ames, Iowa for horse plasma products. Controls received commercially available normal equine plasma prepared from a pool of healthy horses.

### Study populations and experimental infection

Twenty healthy Quarter Horse mare/foal pairs were initially included in this study; 1 unvaccinated mare and her foal were excluded when the foal was stillborn. The unvaccinated group consisted of 7 mare/foal pairs (n = 4 in 2015 and n = 3 in 2016) and the vaccinated group consisted of 12 mare/foal pairs (n = 5 in 2015 and n = 7 in 2016). For the passive infusion of hyperimmune plasma, 9 foals were used, 4 infused with 2 L of commercial normal horse plasma (Immunoglo Serial 1700, Mg Biologics, Ames, IA, USA) and 5 were infused with 2 L of PNAG-hyperimmune plasma produced using standard methods by Mg Biologics. Group assignment was made using a randomized, block design for each year. All foals were healthy at birth and had total serum IgG concentrations >800 mg/dl at 48 h of life using the SNAP Foal IgG test (IDEXX, Inc., Westbrook, Maine, USA), and remained healthy through the day of experimental challenge. Immediately prior to experimental infection with *R*. *equi*, each foal’s lungs were evaluated by thoracic auscultation and thoracic ultrasonography to document absence of pre-existing lung disease.

To study vaccine efficacy, foals were experimentally infected with 1 x 10^6^ of live *R*. *equi* strain EIDL 5–331 (a virulent, *vapA*-gene-positive isolate recovered from a pneumonic foal). This strain was streaked onto a brain-heart infusion (BHI) agar plate (Bacto Brain Heart Infusion, BD, Becton, Dickinson and Company, Sparks, MD, USA). One CFU was incubated overnight at 37°C in 50 ml of BHI broth on an orbital shaker at approximately 240 rpm. The bacterial cells were washed 3 times with 1 X phosphate-buffered saline (PBS) by centrifugation for 10 min, 3000 x g at 4°C. The final washed pellet was resuspended in 40 ml of sterile medical grade PBS to a final concentration of 2.5 x 10^4^ CFU/ml, yielding a total CFU count of 1 x 10^6^ in 40 ml. Half of this challenge dose (20 ml with 5 x 10^5^) was administered transendoscopically to the left mainstem bronchus and the other half (20 ml with 5 x 10^5^) was administered to the right mainstem bronchus. Approximately 200 μl of challenge dose was saved to confirm the concentration (dose) administered, and to verify virulence of the isolate using multiplex PCR (23).

For transendoscopic infection, foals were sedated using intravenous (IV) injection of romifidine (0.8 mg/kg; Sedivet, Boehringer-Ingelheim Vetmedica, Inc., St. Joseph, MO, USA) and IV butorphanol (0.02 mg/kg; Zoetis, Florham Park, New Jersey, USA). An aseptically-prepared video-endoscope with outer diameter of 9-mm was inserted via the nares into the trachea and passed to the bifurcation of the main-stem bronchus. A 40-mL suspension of virulent EIDL 5–331 *R*. *equi* containing approximately 1 x 10^6^ viable bacteria was administered transendoscopically, with 20 ml infused into the right mainstem bronchus and 20 ml into the left mainstem bronchus via a sterilized silastic tube inserted into the endoscope channel. The silastic tube was flushed twice with 20 ml of air after each 20-ml bacterial infusion. Foals and their mares were housed individually and separately from other mare and foal pairs following experimental infection.

### Sample collections from mares and foals

Colostrum was collected (approx. 15 ml) within 8 hours of foaling. Blood samples were collected from immunized mares 6 weeks and 3 weeks before their predicted dates of foaling, and on the day of foaling. Blood samples from 4 non-vaccinated mares in the 2015 study were only collected on the day of foaling, whereas blood was collected from the 3 non-vaccinated mares in the 2016 study at the same time-points as those for vaccinated mares. Blood for preparation of hyperimmune plasma was collected from immunized adult horses 2 weeks after the second injection of 200 μg of the 5GlcNH_2_-TT vaccine plus 0.1 ml of Specol in a total volume of 1 ml.

Blood samples were drawn from foals on day 2 (the day after foaling), and at 4, 6, 8, and 12 weeks of age. Samples at 4 weeks (25–28 days of life) were collected prior to infection. Blood was collected in EDTA tubes for complete blood count (CBC) testing, in lithium heparinized tubes for PBMC isolation, and in clot tubes for serum collection. Transendoscopic tracheobronchial aspiration (T-TBA) was performed at the time of onset of clinical signs for any foals developing pneumonia and at age 12 weeks for all foals (end of study) by washing the tracheobronchial tree with sterile PBS solution delivered through a triple-lumen, double-guarded sterile tubing system (MILA International, Inc. Erlanger, KY, USA).

### Clinical monitoring

From birth until the day prior to infection, foals were observed twice daily for signs of disease. Beginning the day prior to infection, rectal temperature, heart rate, respiratory rate, signs of increased respiratory effort (abdominal lift, flaring nostrils), presence of abnormal lung sounds (crackles or wheezes, evaluated for both hemithoraces), coughing, signs of depressed attitude (subjective evidence of increased recumbence, lethargy, reluctance to rise), and nasal discharges were monitored and results recorded twice daily through 12 weeks (end of study). Thoracic ultrasonography was performed weekly to identify evidence of peripheral pulmonary consolidation or abscess formation consistent with *R*. *equi* pneumonia. Foals were considered to have pneumonia if they demonstrated ≥3 of the following clinical signs: coughing at rest; depressed attitude (reluctance to rise, lethargic attitude, increased recumbency); rectal temperature >39.4°C; respiratory rate ≥60 breaths/min; or, increased respiratory effort (manifested by abdominal lift and nostril flaring). Foals were diagnosed with *R*. *equi* pneumonia if they had ultrasonographic evidence of pulmonary abscessation or consolidation with a maximal diameter of ≥2.0 cm, positive culture of *R*. *equi* from T-TBA fluid, and cytologic evidence of septic pneumonia from T-TBA fluid. The primary outcome was the proportion of foals diagnosed with *R*. *equi* pneumonia. Secondary outcomes included the duration of days meeting the case definition, and the sum of the total maximum diameter (TMD) of ultrasonography lesions over the study period. The TMD was determined by summing the maximum diameters of each lesion recorded in the 4^th^ to the 17^th^ intercostal spaces from each foal at every examination; the sum of the TMDs incorporates both the duration and severity of lesions. Foals diagnosed with *R*. *equi* pneumonia were treated with a combination of clarithromycin (7.5 mg/kg; PO; q 12 hour) and rifampin (7.5 mg/kg; PO; q 12 hour) until both clinical signs and thoracic ultrasonography lesions had resolved. Foals also were treated as deemed necessary by attending veterinarians (AIB; NDC) with flunixin meglumine (0.6 to 1.1 mg/kg; PO; q 12–24 hour) for inflammation and fever.

### Immunoglobulin ELISAs

Systemic humoral responses were assessed among foals by indirectly quantifying concentrations in serum by ELISA from absorbance values of PNAG-specific total IgG and by IgG subisotypes IgG_1_, IgG_4/7_, and IgG_3/5_. ELISA plates (Maxisorp, Nalge Nunc International, Rochester, NY, USA) were coated with 0.6 μg/ml of purified PNAG [43] diluted in sensitization buffer (0.04M PO_4_, pH 7.2) overnight at 4°C. Plates were washed 3 times with PBS with 0.05% Tween 20, blocked with 120 μl PBS containing 1% skim milk for 1 hour at 37°C, and washed again. Mare and foal serum samples were added at 100 μl in duplicate to the ELISA plate and incubated for 1 hour at 37°C. Serum samples were initially diluted in incubation buffer (PBS with 1% skim milk and 0.05% Tween 20) to 1:100 for total IgG titers, 1:64 for IgG_1_ and IgG_4/7_ detection, and to 1:256 for IgG_3/5_ detection. A positive control from a horse previously immunized with the 5GlcNH_2_-TT vaccine and known to have a high titer, along with normal horse serum known to have a low titer, were included in each assay for total IgG titers. For the subisotype assays, immune rabbit serum (rabbit anti-5GLcNH_2_-TT) was diluted to a concentration of 1:102,400 as a positive control and used as the denominator to calculate the endpoint OD ratio of the experimental OD values. The immune rabbit serum was used to account for inter-plate variability and negative control of normal rabbit serum were included with the equine serum samples. After 1 hour incubation at 37°C, the plates were washed 3 times as described above. For total IgG titers, rabbit anti-horse IgG whole molecule conjugated to alkaline phosphatase (Sigma-Aldrich, St. Louis, MO, USA) was used to detect binding. For IgG subisotype detection, 100 μl of goat-anti-horse IgG_4/7_ (Lifespan Biosciences, Seattle, WA, diluted at 1:90,000), or goat anti-horse IgG_3/5_ (Bethyl Laboratories, Montgomery, TX, USA, diluted at 1:30,000) conjugated to horseradish peroxidase, or mouse anti-horse IgG_1_ (AbD Serotec, Raleigh, NC, USA), diluted at 1:25,000) were added to the wells and incubated for 1 hour at room temperature. For the IgG_1_ subisotype, goat antibody to mouse IgG (Bio-Rad, Oxford, England, diluted at 1:1000) conjugated to peroxidase was used for detection. Plates were washed again, and for the total IgG titers pNPP substrate (1 mg/ml) was added while for peroxidase-conjugated antibody to mouse IgG, SureBlue Reserve One Component TMB Microwell Peroxidase Substrate (SeraCare, Gaithersburg, MD, USA) was added to the wells. Plates were incubated for 15 to 60 minutes at 22°C in the dark. The reaction was stopped by adding sulfuric acid solution to the wells. Optical densities were determined at 450 nm by using microplate readers. Equine subisotype concentrations of PNAG-specific IgG_1_, IgG_4/7_, and IgG_3/5_ were also quantified in colostrum of each mare using the same protocol described above for serum. Colostral samples were diluted in incubation buffer (PBS with 1% skim milk and 0.05% Tween 20) to 1:8,192 for IgG_1_, 1:4096 for IgG_4/7_ detection, and at 1:64 for IgG_3/5_ detection. For total IgG endpoint titers were calculated by linear regression using a final OD_405_nm value of 0.5 to determine the reciprocal of the maximal serum dilution reaching this value. For IgG subisotypes, an endpoint OD titer was calculated by dividing the experimental OD values with that achieved by the positive control on the same plate.

### PNAG expression by clinical isolates of *R*. *equi*

Clinical isolates of *R*. *equi* were obtained from the culture collection at the Equine Infectious Disease Laboratory, Texas A&M University College of Veterinary Medicine & Biomedical Sciences. All strains were originally isolated from foals diagnosed with *R*. *equi* pneumonia and were obtained from geographically distinct locations. *R*. *equi* strains were grown overnight on BHI agar then swabbed directly onto glass slides, air dried and fixed by exposure for 1 min to methanol at 4°C. Samples were labeled with either 5 μl of a 5.2 μg/ml concentration of MAb F598 to PNAG directly conjugated to Alexa Fluor 488 or control MAb F429 to alginate, also directly conjugated to Alexa Fluor 488, for 4 hours at room temperature. During the last 5 min of this incubation, 500 nM of Syto83 in 0.5% BSA/PBS pH 7.4 was added to stain nucleic acids (red fluorophore). Samples were washed and mounted for immunofluorescent microscopic examination as described [13].

### Analysis of PNAG expression in infected horse tissues and human monocyte-derived macrophage cultures

The Texas A&M College of Veterinary Medicine & Biomedical Sciences histopathology laboratory provided paraffinized sections of lungs obtained at necropsy from foals with *R*. *equi* pneumonia. Slides were deparaffinized using EzDewax and blocked overnight at 4C with 0.5% BSA in PBS. Samples were washed then incubated with the fluorophore-conjugated MAb F598 to PNAG or control MAb F429 to alginate described above for 4 hours at room temperature. Simultaneously added was a 1:500 dilution (in BSA/PBS) of a mouse antibody to the virulence associated Protein A (VapA) of *R*. *equi*. Binding of the mouse antibody to *R*. *equi* was detected with a donkey antibody to mouse IgG conjugated to Alexa Fluor 555 at a dilution of 1:250 in BSA/PBS. Samples were washed and mounted for immunofluorescence microscopic examination.

To detect PNAG expression in cultured human monocyte-derived macrophages (MDM), prepared as described below in opsonic killing assays, the infected MDM were washed and fixed with 4% paraformaldehyde in PBS for 1 hour at room temperature. To visualize PNAG on the surface of infected cells, MDM cultures were incubated with the fluorophore-conjugated MAb F598 to PNAG or control MAb F429 to alginate for 4–6 hours at room temperature. Samples were then imaged by confocal microscopy to visualize extracellular PNAG expression. Next, these same samples were treated with 100% methanol at 4°C for 5 min at room temperature to permeabilize the plasma membrane. Samples were washed with PBS then incubated with either 5.2 μg/ml of MAb F598 to PNAG or MAb F429 to alginate for 1–2 hours at room temperature, washed in PBS then a 1:300 dilution in PBS of donkey antibody to human IgG labeled with Alexa Fluor 555 added for 4–6 hours at room temperature. Samples were washed and mounted for immunofluorescence microscopic examination.

### C1q deposition assays

An ELISA was used to determine the serum endpoint titers for deposition of equine complement component C1q onto purified PNAG. ELISA plates were sensitized with 0.6 μg PNAG/ml and blocked with skim milk as described above, dilutions of different horse sera added in 50 μl-volumes after which 50 μl of 10% intact, normal horse serum was added as a source of C1q. After 60 minutes incubation at 37°C, plates were washed and 100 μl of goat anti-human C1q, which also binds to equine C1q, diluted 1:1,000 in incubation buffer, added and plates incubated at room temperature for 60 minutes. After washing, 100 μl of rabbit anti-goat IgG whole molecule conjugated to alkaline phosphatase and diluted 1:1,000 in incubation buffer was added and a 1-hour incubation at room temperature carried out. Washing and developing of the color indicator was then carried out as described above, and endpoint titers determined as described above for IgG titers by ELISA.

### Opsonic killing assays

To determine opsonic killing of *R*. *equi*, bacterial cultures were routinely grown overnight at 37°C on chocolate-agar plates, and then killing assessed using modifications of previously described protocols [43]. Modifications included use of EasySep Human Neutrophil Isolation Kits (Stem Cell Technologies Inc., Cambridge, Massachusetts, USA) to purify PMN from blood, and use of gelatin-veronal buffer supplemented with Mg^++^ and Ca^++^ (Boston Bioproducts, Ashland, Massachusetts, USA) as the diluent for all assay components. Final assay tubes contained, in a 400-μl volume, 2 X 10^5^ human PMN, 10% (final concentration) *R*. *equi*-absorbed horse serum as a complement source, 2 X 10^5^ *R equi* cells and the serum dilutions. Tubes were incubated with end-over-end rotation for 90 minutes then diluted in BHI with 0.05% Tween and plated for bacterial enumeration.

For intracellular opsonic killing assays, human monocytes were isolated from peripheral blood using the EasySep Direct Human Monocyte Isolation Kit (Stem Cell Technologies) and 2 x 10^4^ cells placed in a 150 μl volume of RPMI and 10% heat-inactivated autologous human serum in flat-bottom 96-well tissue culture plates for 5–6 days with incubation at 37°C in 5% CO_2_. Differentiated cells were washed and 5 X 10^5^ CFU of either *R*. *equi*, *M*. *avium*, *S*. *aureus*, *N*. *gonorrhoeae*, *L*. *monocytogenes* or *B*. *pertussis*, initially grown on blood or chocolate agar plates at 37°C overnight in 5% CO_2_, suspended in RPMI and 10% heat-inactivated autologous human serum added to the human cells for 30 minutes. Next, these cells were washed and 150 μl of RPMI plus 10% autologous serum with 50 μg gentamicin sulfate/ml added and cells incubated for 24 hours at 37°C in 5% CO_2_. For some experiments, 50 μl of 400 μg/ml of either chitinase (Sigma-Aldrich) or dispersin B (Kane Biotech, Winnipeg, Manitoba), a PNAG-degrading enzyme [31, 44], dissolved in Tris-buffered saline, pH 6.5, were added directly to gentamicin containing wells and plates incubated for 2 hours at 37°C in 5% CO_2_. Cell cultures were washed then combinations of 50 μl of MAb or foal serum, 50 μl of 30% human serum absorbed with the target bacterial strain as a complement source, or heat-inactivated complement as a control, and 50 μl containing 1.5 X 10^5^ human PMN, isolated as described above, added. Controls lacked PMN or had heat-inactivated complement used in place of active complement, and final volumes were made up with 50 μl of RPMI 1640 medium. After a 90-minute incubation at 37°C in 5% CO_2_, 10 μl samples were taken from selected wells for analysis of lysis by lactate dehydrogenase release, and 100 μl of trypsin/EDTA with 0.1% Triton X100 added to all wells lyse the cells via a 10-minute incubation at 37°C. Supernatants were diluted and plated on chocolate or blood agar for bacterial enumeration as described above.

### Cell-mediated immunity

The cell-mediated immune response to vaccination was assessed by measuring IFN-γ production from isolated horse PBMCs that were stimulated with an *R*. *equi* antigen lysate of strain EIDL 5–331, or the same lysate digested for 24 hours at 37°C with 100 μg/ml of dispersin B. The PBMCs were isolated using a Ficoll-Paque gradient separation (GE Healthcare, Piscataway, NJ, USA) and resuspended in 1X RPMI-1640 media with L-glutamine (Gibco, Life Technologies, Grand Island, NY, USA), 15% fetal bovine serum (Gibco), and 1.5% penicillin-streptomycin (Gibco). The PBMCs were cultured for 48 hours at 37°C in 5% CO_2_ with either media only, the mitogen Concanavalin A (positive control; 2.5 μg/ml, Sigma-Aldrich), or *R*. *equi* lysate representing a multiplicity of infection of 10. After 48 hours, supernatants from each group were harvested and frozen at -80°C until examined for IFN-γ production using an equine IFNγ ELISA kit (Mabtech AB, Nacka Strand, Stockholm, Sweden) according to the manufacturer’s instructions. Optical densities were determined using a microplate reader and standard curves generated to determine IFN-γ concentrations in each sample using the Gen 5 software (Biotek, Winooski, VT, USA).

### Statistical methods

Categorical variables with independent observations were compared using chi-squared or, when values for expected cells were ≤5, Fisher’s exact tests. For estimation of the 95% C.I. of the relative risk, the Koopman asymptotic score [27] was determined.

Continuous, independent variables were compared between 2 groups using either paired t-tests or Mann-Whitney tests and between > 2 groups using the Kruskal-Wallis test with pairwise *post hoc* comparisons made using Dunn’s procedure. Continuous variables with non-independent observations (*i*.*e*., repeated measures) were compared using linear mixed-effects modeling with an exchangeable correlation structure and individual mare or foal as a random effect. Survival times were compared non-parametrically using the log-rank test. All analyses were performed using S-PLUS statistical software (Version 8.2, TIBCO, Inc., Seattle, Wash, USA) or the PRISM 7 statistical program. Mixed-effect model fits were assessed using diagnostic residual plots and data were transformed (log_10_) when necessary to meet distributional assumptions of modeling; post hoc pairwise comparisons among levels of a variable (*e*.*g*., age) were made using the method of Sidak [45]. Significance was set at P ≤ 0.05 and adjustment for multiple comparisons made.

## Supporting information

S1 Fig Serum IgG and IgG subisotype titers to PNAG in immunized mare sera. Serum end-point titers of IgG or IgG subisotypes are plotted by vaccine group as a function of age in days. A: Total IgG antibody end-point titers to PNAG were significantly higher in sera of immunized mares at D21 and D0 PF compared with titers in sera of control mares at D0 PF. There was no significant (NS) difference in the IgG titers of the vaccinated mares at pre-immunization and controls at D0 PF. B-D: Concentrations of IgG_1_, IgG_4/7_, and IgG_3/5_ were significantly higher in mares in the vaccinated group at D21 and D0 PF as indicated on the figure. Statistical comparisons were made by linear mixed-effects regression with exchangeable correlation structure, using the mare as random effect (to account for repeated measures) and multiple comparisons determined by the method of Sidak.
(TIFF)

S2 Fig IgG and IgG subisotype titers in mare colostra on day of foaling. End-point colostral titers of IgG or IgG subisotype. End-point values are plotted by vaccine group. A: Total IgG antibody end-point titers to PNAG were significantly higher in colostra of vaccinated mares (N = 10, 2 samples not tested due to limited quantities) compared with colostra of control mares N = 7). B: Concentrations of IgG_1_, IgG_4/7_, and IgG_3/5_ were significantly higher in colostra of mares in the vaccinated group (N = 12). Statistical comparisons were made by the Wilcoxon rank-sum test.
(TIFF)

S3 Fig Antibody titers to PNAG in control and hyperimmune horse plasma infused into newborn foals on day 1 of life. A: Titers of IgG subisotypes and IgA in control (open bars) and PNAG-immune (hatched bars) plasmas. B: horse IgG_1_, C: horse, IgG_3/5_, D: horse IgG_4/7_, or E: horse IgA in sera at day indicated on X-axis. Bars represent medians, error bars the interquartile ranges. OD ratios of IgG_1_, IgG_4/7_, and IgG_3/5_ did not differ significantly over time in controls but were significantly (P < 0.05) greater than age 2 days in foals transfused with anti-PNAG plasma at ages 28, 42, and 56 days (IgG_1_), or ages 28, 42, 56, and 84 for IgG_4/7_ and IgG_3/5_. OD ratio of IgA did not differ significantly (P > 0.05) among the different days for anti-PNAG-transfused foals, but controls had significantly (P < 0.05) higher IgA titers at age 2 days compared to control titers on days 28, 42, and 56. The OD ratio values for control foals’ IgA on day 84 was significantly (P < 0.05) greater than those of control foals on days 28, 42, and 56. IgA titers between controls and anti-PNAG-transfused foals differed significantly (P < 0.05) at day 84 only. All P values were determined by linear mixed-effects regression. F: Opsonic killing of *R*. *equi* EIDL 990 by antibody in control or immune plasma. Monoclonal antibodies (MAb) were used as controls, as were tubes lacking PMN or complement (C’) as indicated. Bars represent means of technical replicates.
(TIFF)

S4 Fig PNAG expression by *R. equi* clinical isolates. Designated individual clinical isolates of *R. equi* were reacted with either control MAb F429 to *P*. *aeruginosa* alginate or MAb F598 to PNAG, both directly conjugated to Alexa Fluor 488. Binding to PNAG on bacterial cells was visualized by immunofluorescence microscopy. Left-hand panel in each pair shows DNA visualized by red-fluorophore Syto 83. Right-hand panel in each pair is green if PNAG detected by MAb F598.
(TIFF)

S5 Fig Expression of PNAG in lungs of *R. equi* infected foals. Either an uninfected control lung or lungs from foals with *R*. *equi* pneumonia were reacted with the indicated antibody to detect the presence of *R*. *equi* (red, antibody to VapA protein), PNAG (Green, MAb F598) or control MAb F429 to alginate. A: Low power (40X) sections indicating presence of *R*. *equi* and closely associated PNAG in infected lung. Bars = 10 μm. B: Higher magnification (60 X) shows individual infected cells in 2 different foal lung sections with PNAG-expressing *R*. *equi* contained in apparent intracellular vesicles.
(TIFF)

S6 Fig Surface and intracellular expression of PNAG in infected human MDM. Detection of PNAG either on the surface or within the infected cell was determined by first reacting cultures with MAb F598 to PNAG or control MAb F429, both directly conjugated to Alexa Fluor 488 (green fluorophore), on paraformaldehyde-fixed cells then washing and permeabilizing the cells with ice-cold methanol followed by reaction with the MAbs and secondary antibody to human IgG conjugated to Alexa Fluor 555 (red/orange). A-G: Cells and treatments indicated in figure. White bars = 10 μm.
(TIFF)

S7 Fig Human MDM cells infected with PNAG-expressing intracellular pathogens have high levels of the PNAG antigen on their surface that is removed by treatment with Dispersin B. PNAG on infected cell surfaces was detected by reacting cultures with MAb F598 to PNAG or control MAb F429, both directly conjugated to Alexa Fluor 488 (green fluorophore), on paraformaldehyde-fixed cells. Infected bacterial strains and treatments indicated in figure. For each figure, upper left quadrant shows nuclear DNA (red), upper right quadrant shows PNAG (green if present), lower left quadrant shows phase contrast, lower right quadrant shows co-localization of DNA and PNAG (yellow-green to yellow to orange if present). White bars = 10 μm.
(TIFF)

S8 Fig Opsonic killing of intracellular pathogens by antibody to PNAG, complement (C’) and PMN depends on infected-cell surface expression of PNAG. A-E: Killing of intracellular bacteria by antibody, PMN and C’ was markedly reduced following treatment of infected cells with Dispersin B (open bars) to digest surface PNAG compared with treatment with the control enzyme, Chitinase (black bars). Depicted data are representative of 2–3 independent experiments. Bars represent means of 6 technical replicates. Bars showing <0% kill represent data wherein the cfu counts were greater than the control of PNAG MAb + PMN + HI C’.
(TIFF)

S9 Fig Release of LDH from cells infected with intracellular pathogens following antibody plus immune effector treatment. A and B: Replicate experiments measuring release of LDH from cells infected with indicated pathogen treated with 10 μg/ml control or anti-PNAG monoclonal or 10% polyclonal antibody plus indicated immune effector. Bars indicate means of quadruplicates. C: Summary of LDH release for experiment in figure S9B. Mean (bars) and 95% C.I. (error bars) indicate release of LDH from cells infected with all five intracellular pathogens. Overall ANOVA P value by one-way repeated measures ANOVA, pair wise comparisons determined by two-stage linear step-up procedure of Benjamini, Krieger and Yekutieli.
(TIFF)
