# Supplementary figures and images for: Antibody to Poly-N-acetyl glucosamine provides protection against intracellular pathogens: Mechanism of action and validation in horse foals challenged with Rhodococcus equi
Source: PLoS Pathog. 2018 Jul 19;14(7):e1007160. doi: 10.1371/journal.ppat.1007160 (PMC6053243; doi:10.1371/journal.ppat.1007160)

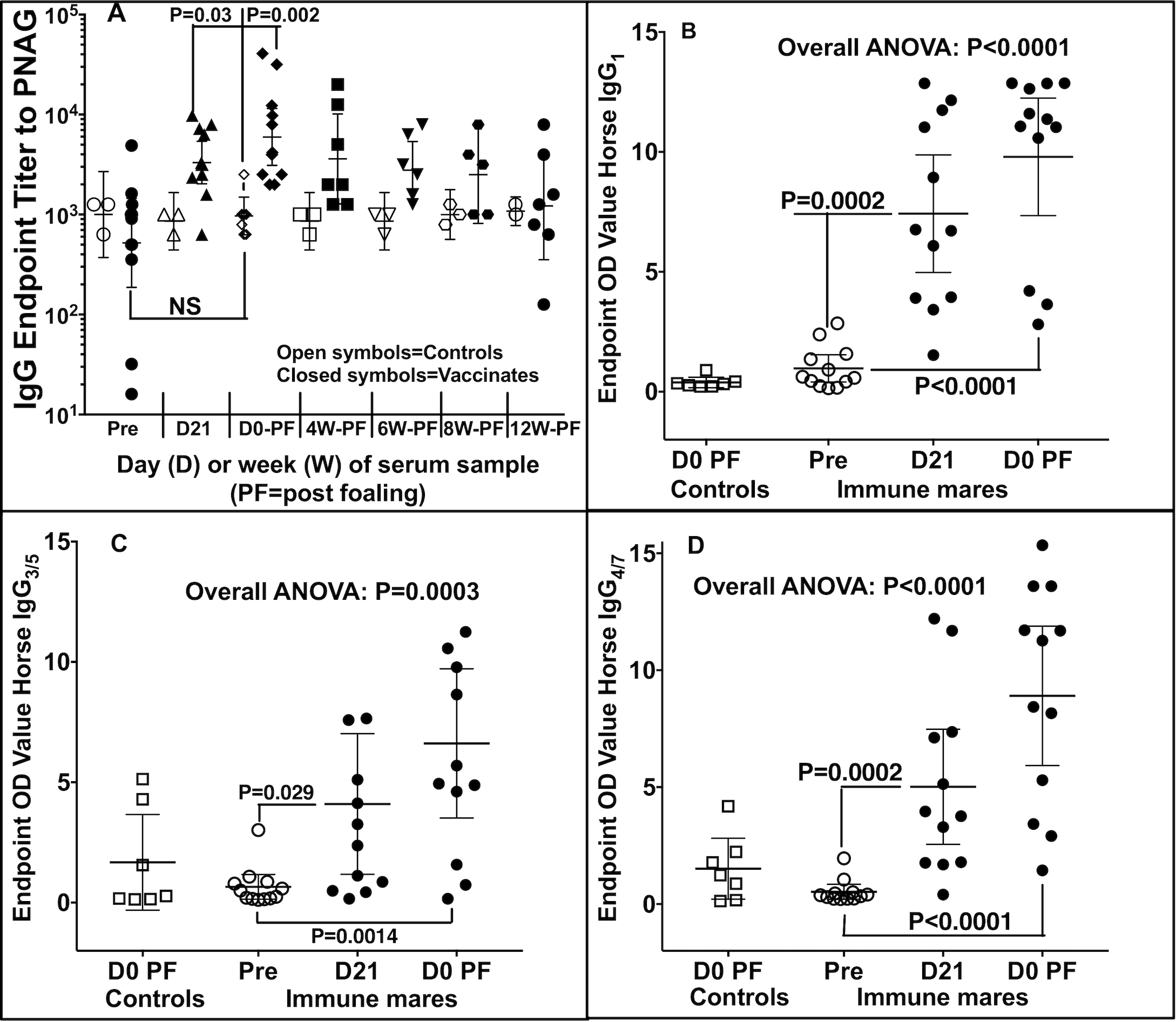

Supplement: S1 Fig — Serum end-point titers of IgG or IgG subisotypes are plotted by vaccine group as a function of age in days. A: Total IgG antibody end-point titers to PNAG were significantly higher in sera of immunized mares at D21 and D0 PF compared with titers in sera of control mares at D0 PF. There was no significant (NS) difference in the IgG titers of the vaccinated mares at pre-immunization and controls at D0 PF. B-D: Concentrations of IgG1, IgG4/7, and IgG3/5 were significantly higher in mares in the vaccinated group at D21 and D0 PF as indicated on the figure. Statistical comparisons were made by linear mixed-effects regression with exchangeable correlation structure, using the mare as random effect (to account for repeated measures) and multiple comparisons determined by the method of Sidak. (TIFF) [file ppat.1007160.s001.tiff]

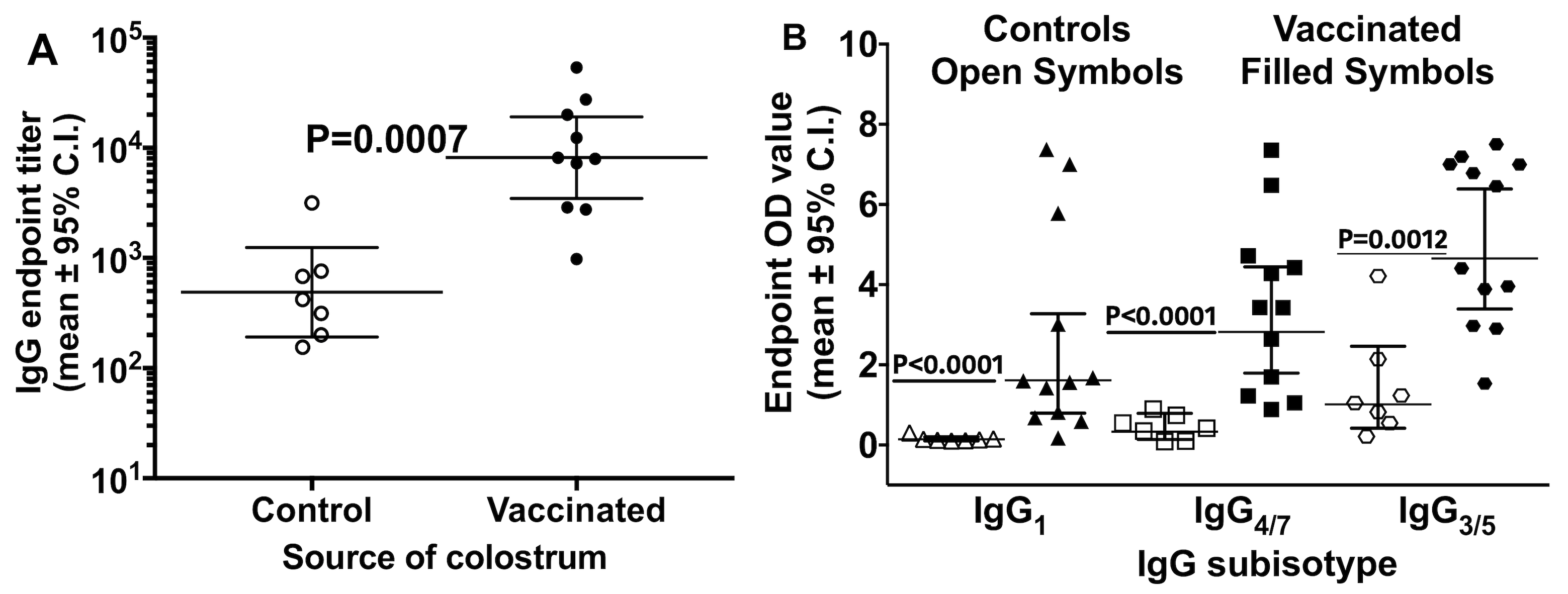

Supplement: S2 Fig — End-point colostral titers of IgG or IgG subisotype. End-point values are plotted by vaccine group. A: Total IgG antibody end-point titers to PNAG were significantly higher in colostra of vaccinated mares (N = 10, 2 samples not tested due to limited quantities) compared with colostra of control mares N = 7). B: Concentrations of IgG1, IgG4/7, and IgG3/5 were significantly higher in colostra of mares in the vaccinated group (N = 12). Statistical comparisons were made by the Wilcoxon rank-sum test. (TIFF) [file ppat.1007160.s002.tiff]

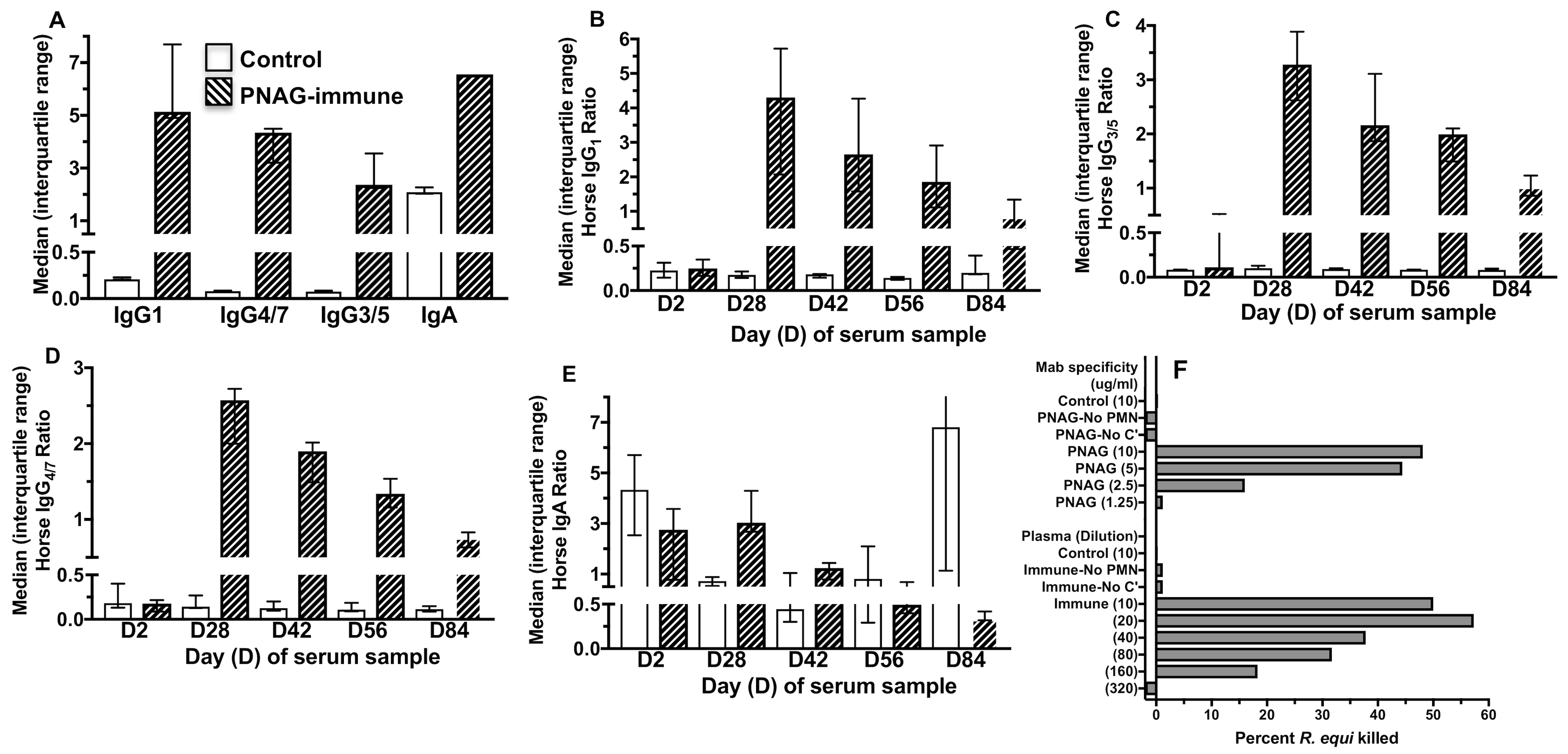

Supplement: S3 Fig — A: Titers of IgG subisotypes and IgA in control (open bars) and PNAG-immune (hatched bars) plasmas. B: horse IgG1, C: horse, IgG3/5, D: horse IgG4/7, or E: horse IgA in sera at day indicated on X-axis. Bars represent medians, error bars the interquartile ranges. OD ratios of IgG1, IgG4/7, and IgG3/5 did not differ significantly over time in controls but were significantly (P < 0.05) greater than age 2 days in foals transfused with anti-PNAG plasma at ages 28, 42, and 56 days (IgG1), or ages 28, 42, 56, and 84 for IgG4/7 and IgG3/5. OD ratio of IgA did not differ significantly (P > 0.05) among the different days for anti-PNAG-transfused foals, but controls had significantly (P < 0.05) higher IgA titers at age 2 days compared to control titers on days 28, 42, and 56. The OD ratio values for control foals’ IgA on day 84 was significantly (P < 0.05) greater than those of control foals on days 28, 42, and 56. IgA titers between controls and anti-PNAG-transfused foals differed significantly (P < 0.05) at day 84 only. All P values were determined by linear mixed-effects regression. F: Opsonic killing of R. equi EIDL 990 by antibody in control or immune plasma. Monoclonal antibodies (MAb) were used as controls, as were tubes lacking PMN or complement (C’) as indicated. Bars represent means of technical replicates. (TIFF) [file ppat.1007160.s003.tiff]

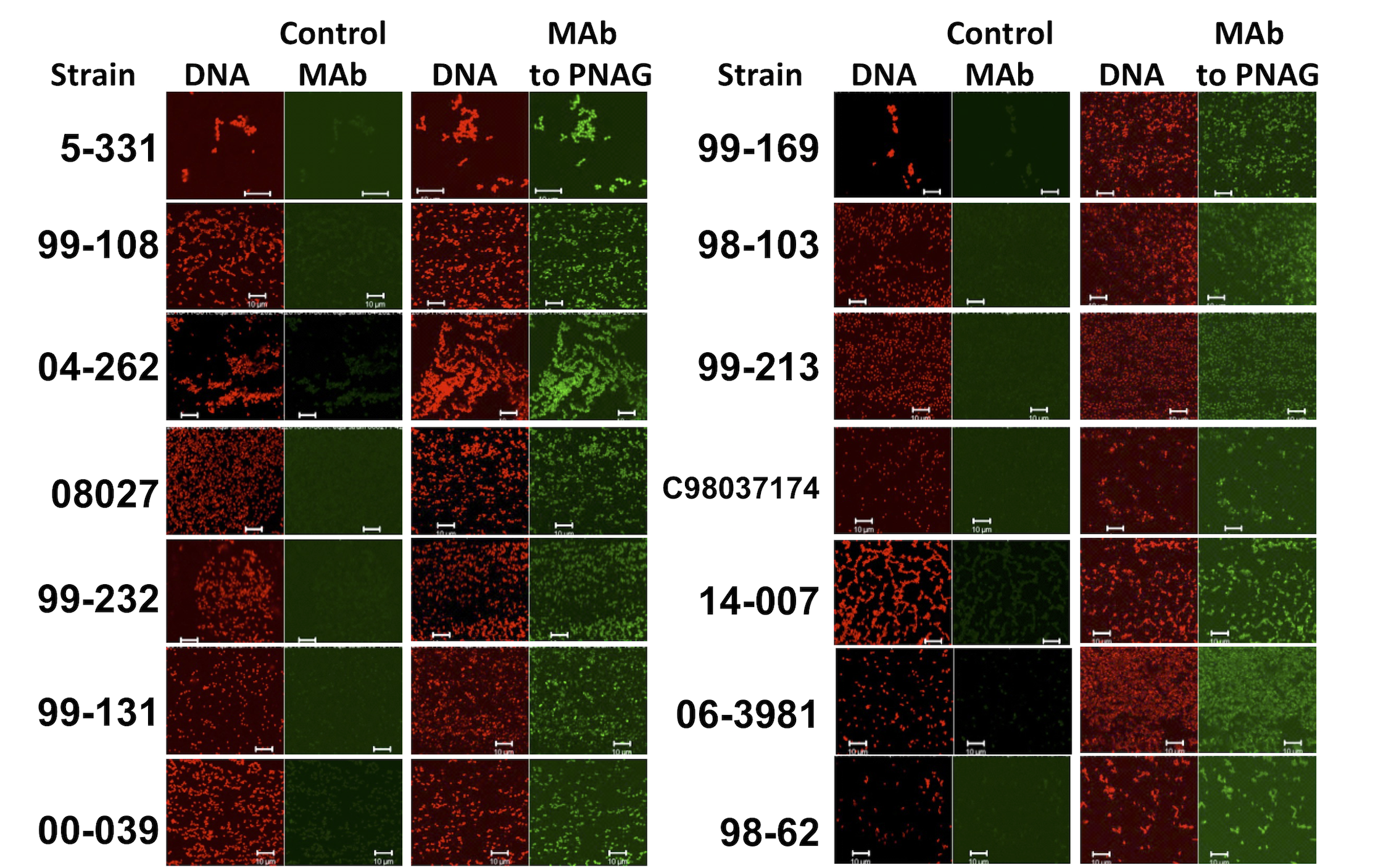

Supplement: S4 Fig — Designated individual clinical isolates of R. equi were reacted with either control MAb F429 to P. aeruginosa alginate or MAb F598 to PNAG, both directly conjugated to Alexa Fluor 488. Binding to PNAG on bacterial cells was visualized by immunofluorescence microscopy. Left-hand panel in each pair shows DNA visualized by red-fluorophore Syto 83. Right-hand panel in each pair is green if PNAG detected by MAb F598. (TIFF) [file ppat.1007160.s004.tiff]

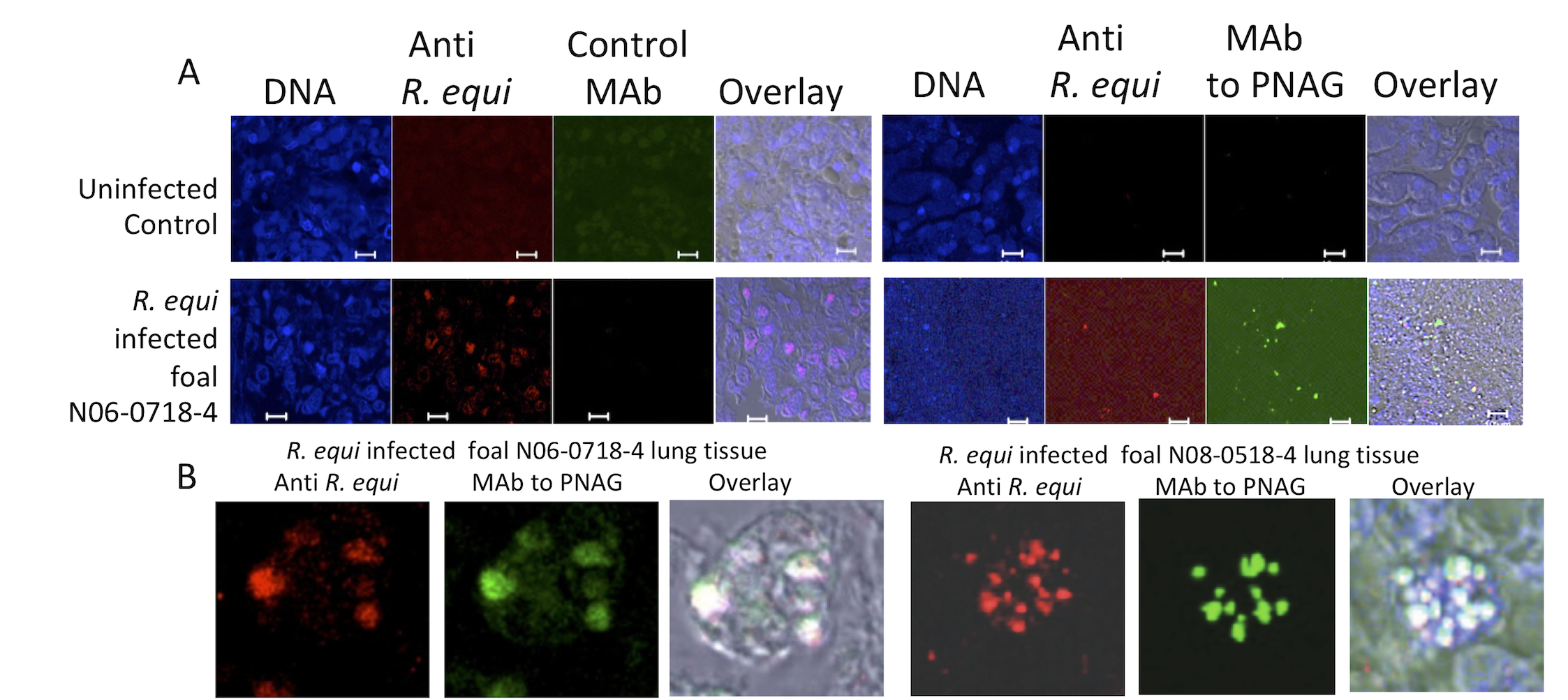

Supplement: S5 Fig — Either an uninfected control lung or lungs from foals with R. equi pneumonia were reacted with the indicated antibody to detect the presence of R. equi (red, antibody to VapA protein), PNAG (Green, MAb F598) or control MAb F429 to alginate. A: Low power (40X) sections indicating presence of R. equi and closely associated PNAG in infected lung. Bars = 10 μm. B: Higher magnification (60 X) shows individual infected cells in 2 different foal lung sections with PNAG-expressing R. equi contained in apparent intracellular vesicles. (TIFF) [file ppat.1007160.s005.tiff]

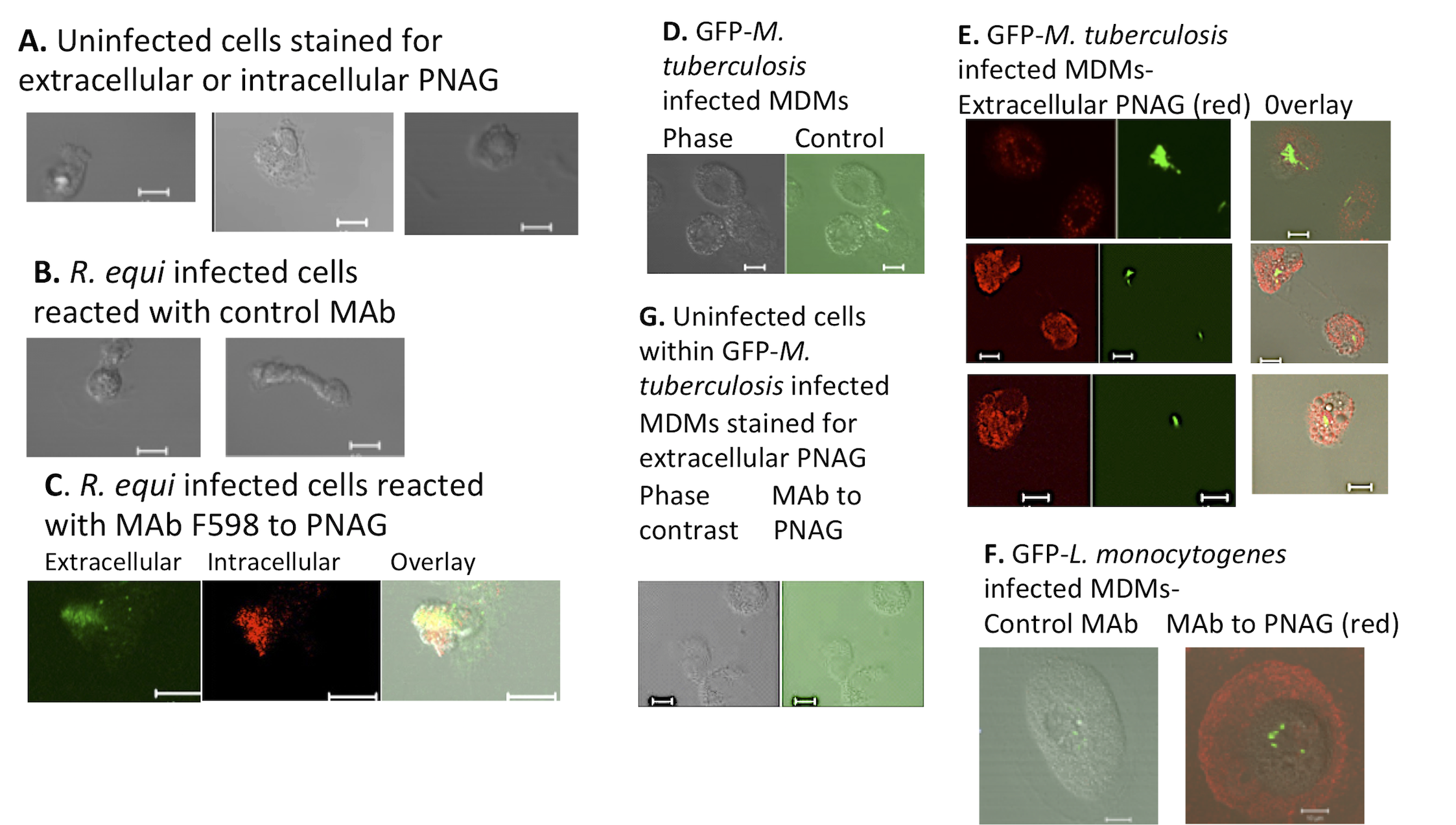

Supplement: S6 Fig — Detection of PNAG either on the surface or within the infected cell was determined by first reacting cultures with MAb F598 to PNAG or control MAb F429, both directly conjugated to Alexa Fluor 488 (green fluorophore), on paraformaldehyde-fixed cells then washing and permeabilizing the cells with ice-cold methanol followed by reaction with the MAbs and secondary antibody to human IgG conjugated to Alexa Fluor 555 (red/orange). A-G: Cells and treatments indicated in figure. White bars = 10 μm. (TIFF) [file ppat.1007160.s006.tiff]

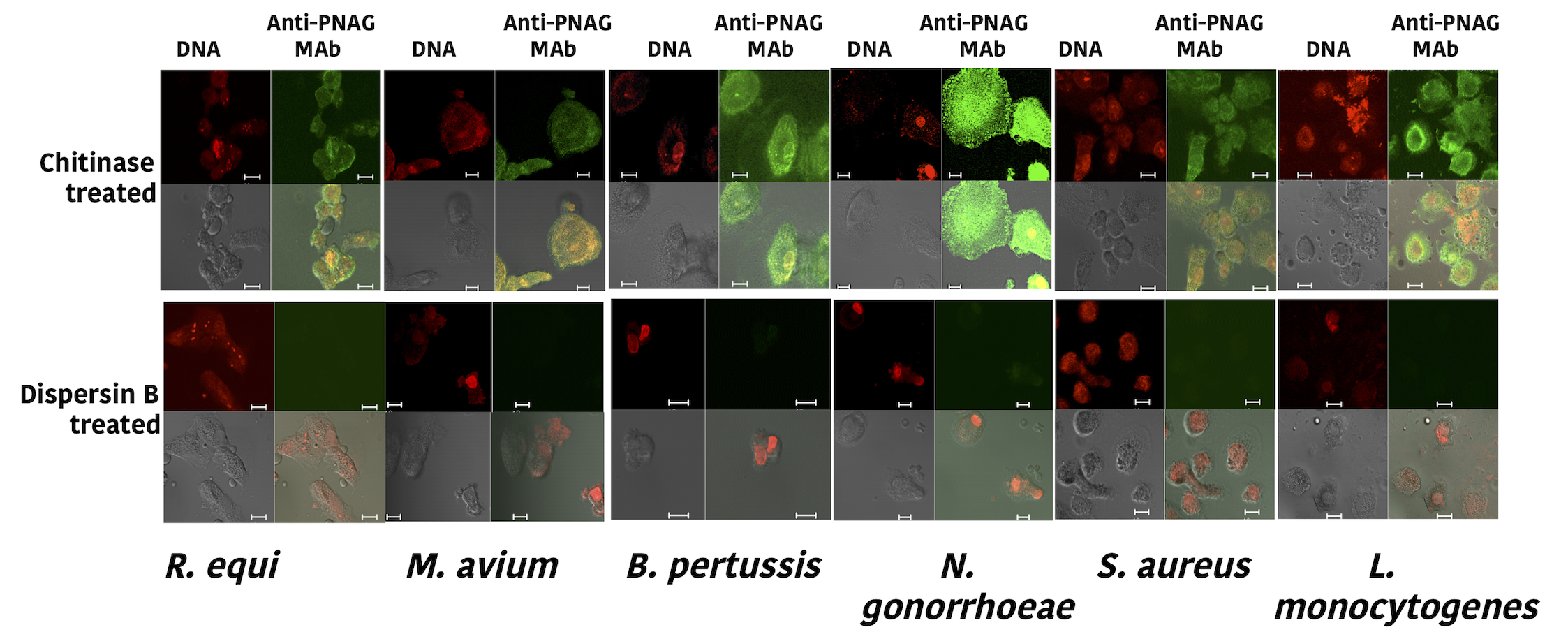

Supplement: S7 Fig — PNAG on infected cell surfaces was detected by reacting cultures with MAb F598 to PNAG or control MAb F429, both directly conjugated to Alexa Fluor 488 (green fluorophore), on paraformaldehyde-fixed cells. Infected bacterial strains and treatments indicated in figure. For each figure, upper left quadrant shows nuclear DNA (red), upper right quadrant shows PNAG (green if present), lower left quadrant shows phase contrast, lower right quadrant shows co-localization of DNA and PNAG (yellow-green to yellow to orange if present). White bars = 10 μm. (TIFF) [file ppat.1007160.s007.tiff]

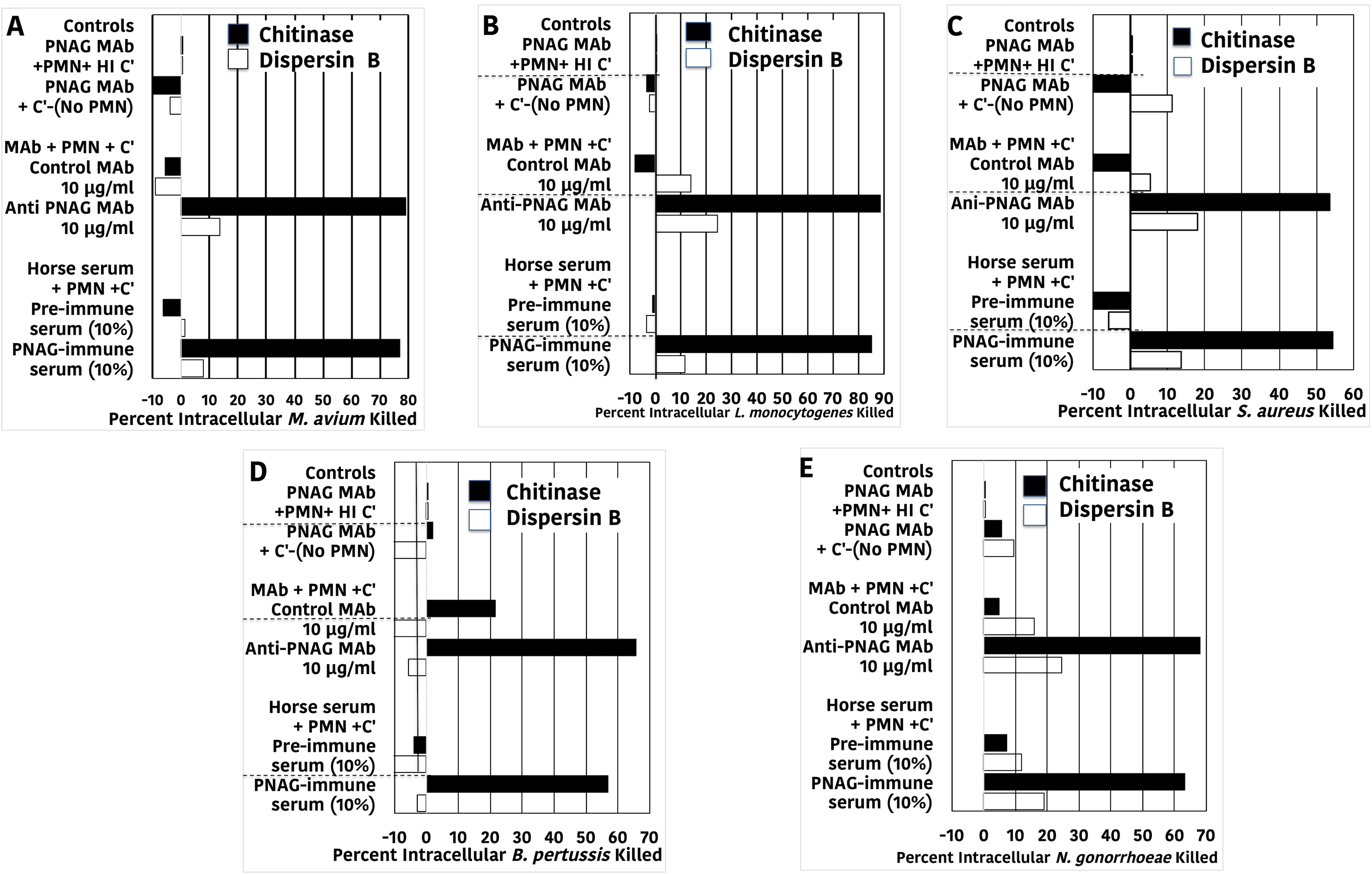

Supplement: S8 Fig — A-E: Killing of intracellular bacteria by antibody, PMN and C’ was markedly reduced following treatment of infected cells with Dispersin B (open bars) to digest surface PNAG compared with treatment with the control enzyme, Chitinase (black bars). Depicted data are representative of 2–3 independent experiments. Bars represent means of 6 technical replicates. Bars showing <0% kill represent data wherein the cfu counts were greater than the control of PNAG MAb + PMN + HI C’. (TIFF) [file ppat.1007160.s008.tiff]

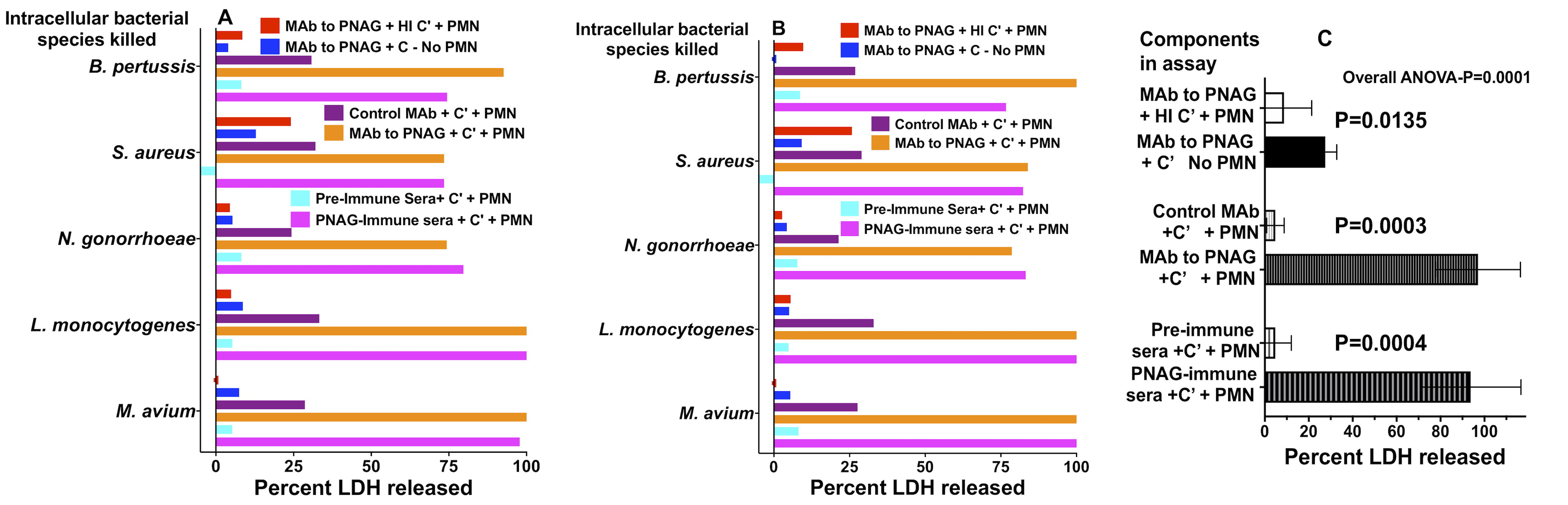

Supplement: S9 Fig — A and B: Replicate experiments measuring release of LDH from cells infected with indicated pathogen treated with 10 μg/ml control or anti-PNAG monoclonal or 10% polyclonal antibody plus indicated immune effector. Bars indicate means of quadruplicates. C: Summary of LDH release for experiment in figure S9B. Mean (bars) and 95% C.I. (error bars) indicate release of LDH from cells infected with all five intracellular pathogens. Overall ANOVA P value by one-way repeated measures ANOVA, pair wise comparisons determined by two-stage linear step-up procedure of Benjamini, Krieger and Yekutieli. (TIFF) [file ppat.1007160.s009.tiff]
